# Abnormal Wnt and PI3Kinase Signaling in the Malformed Intestine of *lama5* Deficient Mice

**DOI:** 10.1371/journal.pone.0037710

**Published:** 2012-05-30

**Authors:** Léa Ritié, Caroline Spenlé, Joël Lacroute, Anne-Laure Bolcato-Bellemin, Olivier Lefebvre, Christine Bole-Feysot, Bernard Jost, Annick Klein, Christiane Arnold, Michèle Kedinger, Dominique Bagnard, Gertraud Orend, Patricia Simon-Assmann

**Affiliations:** 1 Inserm, U682, Strasbourg, France; Univ de Strasbourg, France; 2 Polyplus-transfection, Illkirch, France; 3 Microarray and Sequencing Platform, IGBMC, Illkirch, France; Northwestern University Feinberg School of Medicine, United States of America

## Abstract

Laminins are major constituents of basement membranes and are essential for tissue homeostasis. Laminin-511 is highly expressed in the intestine and its absence causes severe malformation of the intestine and embryonic lethality. To understand the mechanistic role of laminin-511 in tissue homeostasis, we used RNA profiling of embryonic intestinal tissue of *lama*5 knockout mice and identified a *lama5* specific gene expression signature. By combining cell culture experiments with mediated knockdown approaches, we provide a mechanistic link between laminin α5 gene deficiency and the physiological phenotype. We show that laminin α5 plays a crucial role in both epithelial and mesenchymal cell behavior by inhibiting Wnt and activating PI3K signaling. We conclude that conflicting signals are elicited in the absence of *lama5*, which alter cell adhesion, migration as well as epithelial and muscle differentiation. Conversely, adhesion to laminin-511 may serve as a potent regulator of known interconnected PI3K/Akt and Wnt signaling pathways. Thus deregulated adhesion to laminin-511 may be instrumental in diseases such as human pathologies of the gut where laminin-511 is abnormally expressed as it is shown here.

## Introduction

Development and homeostasis of the mammalian intestine is a complex morphogenetic process that requires sequential interactions between cells and the extracellular matrix (ECM). Inductive interactions between gut endoderm and the underlying mesenchyme pattern the developing digestive tract into regions with specific morphology and function. Specification into distinct regions involves transcription factors such as the Hox and caudal family of molecules [1], [2]. Cellular proliferation and differentiation in the intestine depends also on a multitude of different signals [2], [3]. In particular, the Wnt signaling plays a critical role in normal development, adult homeostasis, and tumorigenesis of the intestine [3], [4].

ECM molecules and in particular BM components shape the sequential and reciprocal interaction between the epithelium and the mesenchyme. The BM creates a combination of permissive and inhibitory cues and its composition is regulated in space and time. In the intestine, the laminin family of glycoproteins represents a major component of the BM found in the interface between endoderm and mesenchyme in the embryo and between epithelial cells and the underlying connective tissue in the adult tissue [5], [6]. Laminins regulate processes including cell adhesion, migration, angiogenesis, differentiation, tumor growth and metastasis [7]. Laminins contain a single α-, β- and γ-chain that assemble into a cross-shaped trimer and form 16 different isoforms [8]. Laminin-511 (α5β1γ1) is the prominent α5-containing laminin isoform (**Figure S1**) in the epithelial BM of developing and adult organs including intestine and is also found around individual smooth muscle cells [9]–[11]. The effects of laminins on cellular behavior depend on the receptors that participate in intracellular signaling, namely β1- and β4-integrins, the dystroglycan complex and the Lutheran-glycoprotein [8].

A mouse lacking LMα5 gene expression is embryonic lethal (E17) which suggests an essential role of laminin-511 in embryonic development [12]. Knockout embryos exhibit multiple tissue defects, including exencephaly, abnormalities in craniofacial anatomy, lung, kidney, tooth and hair follicle development as well as alterations in neural crest cell migration [12]–[17]. In the intestine, we showed that the LMα5 chain plays a crucial role in the process of embryonic intestinal folding during development of the musculature (**Figure S1**), and on the mucus epithelial cell lineage [18]. More recently, using a knockout and transgenic-rescue strategy, it was shown that reduced LMα5 expression and concomitant elevated expression of laminins α1 and α4 in the subepithelial BM of the small intestine was linked to transformation of the small intestine into a tissue resembling the colonic mucosa [19].

Our knowledge about the role of laminins in gastrointestinal pathologies is very limited. Alterations of laminin expression are detected in the small intestine of Crohn's disease patients and of children affected by intractable diarrhea called tufting enteropathy [20], [21]. In Hirschsprung disease, a developmental disorder that is associated with failure of enteric ganglia formation, an alteration in laminin expression including the α5 chain was noted in muscle layers and myenteric ganglia [22]. Thus it is possible that laminin-511, the major α5-containing laminin isoform of the intestine, is instrumental in activating signaling that is crucial in development, tissue homeostasis and human intestinal pathologies.

Little is known about the downstream targets of ECM components *in vivo*. Specific inactivation of BM molecules in mice combined with microarray analysis should help to investigate the intracellular signal transduction cascades activated upon contact of cells with a particular ECM molecule. The goal of our study was to elucidate how the α5 chain-containing laminins affect intestinal organogenesis and cell behavior. For this purpose we used the expression profiling technology to define signaling pathways that may underlie cell behavior on laminin-511 in its context of intestinal tissue organogenesis. We used a targeted LMα5 knockout mouse model and small *lama*5 interfering RNAs. We showed that laminin-511 is essentially required for survival, epithelial morphogenesis and differentiation. Our data provide evidence for a link of signaling by laminin-511 to activation of PI3K/Akt and inhibition of Wnt signaling.

## Results

### Lack of the LMα5 chain has a profound effect on the intestinal gene expression signature

**Figure 1:**
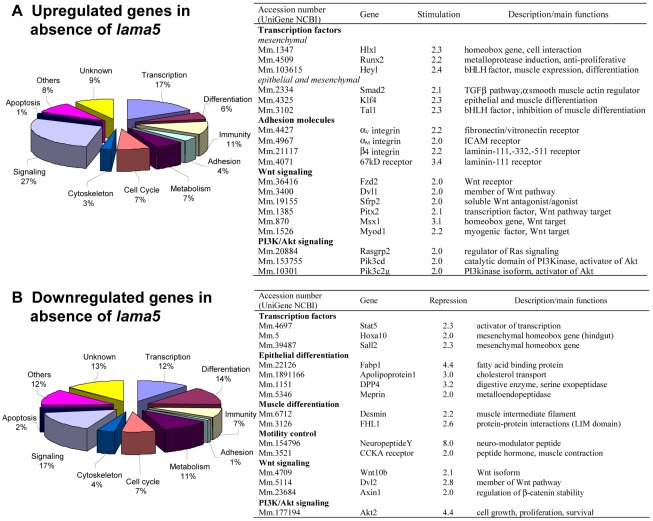
Pie chart of upregulated and downregulated genes in the absence of *lama5.* The 192 upregulated and the 164 downregulated genes were grouped according to their presumed function. Brief description and main functions of deregulated genes in *lama5* knockout intestines are given as well as the rate of altered expression.

To address the role of the LMα5 chain during organogenesis and cell interactions, a microarray analysis was performed. RNA was extracted from whole E-15.5 intestines of LMα5-deficient and wildtype mice. Embryonic day 15.5 was chosen since at this time point villus morphogenesis and differentiation of smooth muscle are initiated [2]. Analysis of the RNA expression profiling revealed that 192 genes are upregulated and 164 genes are downregulated more than 2-fold in LMα5- deficient intestine in comparison to wild-type tissue (**Figure S2**). These differentially expressed genes were classified according to their presumed functions (**Figure 1**). Amongst the upregulated genes, 27% of genes (51 genes) are involved in signal transduction such as the Wnt and PI3K/Akt pathways. Moreover, 17% of genes (32 genes) encode molecules implied in gene transcription with some transcription factors relevant in epithelial or in mesenchymal tissue development and homeostasis. Furthermore, 4% of genes belong to the adhesion receptor family including three integrin subunits (αv, αM and β4) and the 67 kd laminin-111 receptor. Amongst the downregulated genes, again signaling molecules are the most affected (17%). 14% of genes (23 genes) are associated with epithelial or muscle cell differentiation (**Figure 1** **, Table S1**).

### Laminin-511 regulates expression of genes involved in epithelial cell adhesion and differentiation

Integrins and Lutheran are amongst the cell adhesion receptors that interact with LMα5-containing trimeric matrix molecules. Here we found that expression of the β4 integrin is increased at the mRNA (**Figure 1**) and protein level (**Figure S3**). In contrast β1 integrin expression is already low in controls and is slightly further decreased in the absence of *lama5* (**Figure S3**). By tissue staining we saw that Lutheran is strongly decreased in the intestine as well as in the lung anlagen of LMα5 deficient mice (**Figure S3**).

Several genes encoding markers of epithelial differentiation are downregulated in the absence of the LMα5 chain (**Figure 1**). These include molecules involved in lipid/cholesterol metabolism such as Fabp1 and Fabp2, ApoA1 and HmgCs2. By semi-quantitative RT-PCR we investigated their expression and confirmed the decrease of these transcripts in intestinal tissue from LMα5 deficient mice in comparison to wildtype littermates (**Figure S4**). The expression levels (1.5-to 3.1-fold decrease) are similar to those obtained by the microarray analysis. The absence of the LMα5 chain also causes a reduced expression of genes encoding brush border enzymes such as the zinc metalloprotease Mep1a (Meprin) and the serine exopeptidase DPP4 (**Figure 1**).

### Myogenic differentiation markers are affected by the lack of LMα5

**Figure 2:**
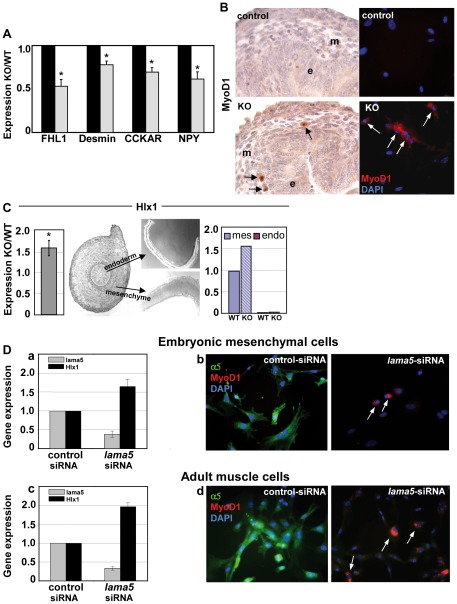
Muscle differentiation genes are regulated by laminin α5 chain. (A) Semi-quantitative RT-PCR experiments were performed on E15.5 control and knockout intestines for genes belonging to the muscle compartment. Data are presented as fold changes between knockout (grey bars) and wild-type (black bars) (mean +/− SEM, n = 5 to n = 9) (* p<0.05). (B) Immunostaining of MyoD1 on E15.5 control and KO intestines or on derived-cultured mesenchymal cells shows that MyoD1 is induced in knockout mesenchymal cells (arrows). Nuclei are stained with DAPI (blue). (C) Expression of Hlx1 by RT-qPCR showing that its expression is enhanced in *lama5* deficient versus wild-type intestines (mean +/− SEM, n = 6) (* p<0.05). Quantitative RT-PCR was performed on separated endodermal and mesenchymal compartments. The diagram shows the relative expression of Hlx1 between mesenchyme (mes) and endoderm (endo) with value 1 representing the total amount in wild-type intestines. Expression of Hlx1 is increased specifically in the mesenchymal compartment of LMα5^−/−^ intestines. (D) Effect of *lama5* siRNA on mesenchyme-derived target gene expression: Hlx1 (a, c) and MyoD1 (b, d). Embryonic mesenchymal cells (panels a and b) and adult intestinal smooth muscle cells (panels c and d) were cultured in the presence of control- and *lama5*-siRNA, respectively. *Lama5*-siRNA decreases LMα5 gene (up to 68%) and protein expression (in green) in both embryonic and adult cells. Note that *lama5*-siRNA upregulates Hlx1 gene expression and MyoD protein expression. After 72 h, gene expression was analyzed by RT-qPCR upon normalization to GAPDH and is expressed as relative fold-change (mean +/− SEM; n = 3) compared to control-siRNA. Arrows point at MyoD positive cells. Nuclei are stained with DAPI.

In accordance with the observation that the LMα5 deficient intestine displays a smooth muscle defect [18], we find a repression of genes regulating the mesenchymal and muscle compartment. In particular gene products regulating gut motility such as FHL1 (a regulator of muscle cell differentiation), desmin as well as NPY and CCKAR are downregulated in LMα5 deficient intestinal tissue (**Figure 1**). The downregulation was confirmed by semi-quantitative RT-PCR (**Figure 2A**) and the reduced expression of the muscle marker desmin is in agreement with our already published data at protein level [18].

Unexpectedly, our microarray experiments revealed an upregulation of MyoD, a classical sketelal muscle-specific transcription factor, and of Hlx1, known to be required for smooth muscle cell differentiation, in absence of the *lama5* gene (**Figure 1**). Upregulation of MyoD1 was confirmed by immunostaining that revealed a MyoD1-positive signal in E15.5 α5 knockout intestine as well as in cultured intestinal mesenchymal cells derived from LMα5 deficient embryonic intestines (**Figure 2B**). Such sporadic MyoD-positive myoblasts were described *in vivo* in the adult intestine [23]. The increased expression of Hlx1 in absence of the LMα5 chain is confined to the mesenchymal compartment as confirmed by RT-qPCR on RNA derived from isolated embryonic intestinal endoderm and mesenchyme (**Figure 2C**).

To determine whether laminin-511 is necessary to regulate expression of the identified target genes, we used siRNA to downregulate *lama5* in wild-type embryonic mesenchymal cells and adult intestinal smooth muscle cells, which reached 60% and 68% repression in the embryonic and adult cells, respectively as shown by RT-qPCR (**Figure 2D** **a, c**) and immunofluorescence (**Figure 2D** **, b, d**). Analysis of Hlx1 gene expression by RT-qPCR and of MyoD1 protein by immunofluorescence showed a 1.7-fold increase of Hlx1 and the appearance of MyoD1-positive nuclei upon silencing of *lama5* in cells of embryonic and adult origin (**Figure 2D**).

### Laminin-511 inhibits canonical Wnt signaling

**Figure 3:**
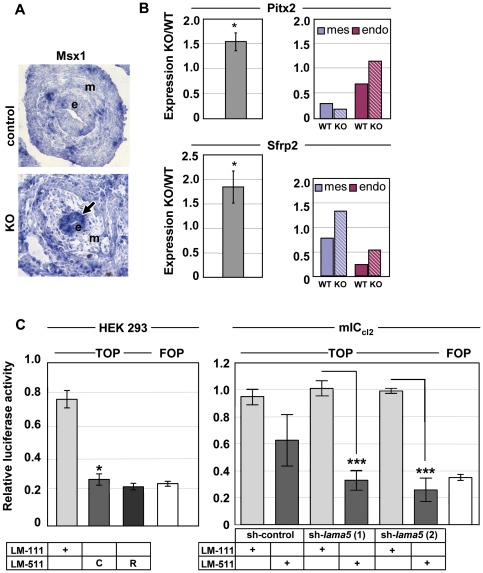
Presence of laminin-511 inhibits TOPflash activity. (A) *In situ* hybridization of Msx1 on embryonic control and KO intestines showing that Msx1 is stimulated in knockout endodermal cells (arrow); e: endoderm; m: mesenchyme. (B) Gene expression ratios determined by RT-qPCR of Pitx2 and Sfrp2 between intestinal E15.5 knockout and control tissues, and on isolated mesenchymal or endodermal compartments confirm the increase of both molecules in the absence of laminin α5; for further details see legend to figure 2 (mean +/− SEM, n = 7–9; * p<0.02). (C) HEK293 cells and lentiviral *lama5* shRNA m-IC_Cl2_ infected cells seeded on plastic, laminin-111, cell-derived laminin-511 (LM-511C) or on recombinant laminin-511(LM-511R) were transfected with TOPflash or the negative FOPflash vector. The graphs represent the average relative luciferase activity normalized to luciferase Renilla activity; this ratio was then normalized to that obtained on plastic (n = 5, n = 3 for HEK293 on laminin-111, n = 1 for laminin-511(R); in duplicate; mean +/− SEM). For each cell line, TOPflash activity on laminin-111 does not statistically differ to that observed on plastic. Note that the TOPflash activity is statistically inhibited when cells are grown on laminin-511 as compared to laminin-111 (* p<0.05; *** p<0.001).

The Wnt/β-catenin signaling pathway is implicated in the development and homeostasis of almost all organs including the intestine [24], [25]. In this pathway, positive and negative regulation is integrated at level of β-catenin stabilization and impacts on of target gene expression. The absence of *lama5* in mouse had an influence on the expression of several Wnt genes such as axin1, Dvl2, Wnt10b that are downregulated while in contrast Dvl1, Fzd2, sFRP2 are upregulated (**Figure 1**). However, expression of some other Wnt genes known to be expressed in the embryonic murine intestine such as Wnt4, Wnt5a and Wnt11 [26] were unchanged (not shown). Expression of four Wnt target genes – MyoD1, Hlx, Msx1, Pitx2 – (“the Wnt home page”; [27]) is upregulated in intestinal tissue lacking *lama5* (**Figure 1**). *In situ* hybridization and RT-qPCR allowed us to confirm the upregulation of some of these genes such as Msx1 (**Figure 3A**), Pitx2 and Sfrp2 (**Figure 3B**), MyoD and Hlx1 (**Figure 2B and C**) in the α5 knockout intestine in comparison to the wild-type tissue.

Expression of Pitx2 and Sfrp2 was previously reported in the fetal intestine [28]. Here we could show that although Pitx2 and Sfrp2 are expressed in both endodermal and mesenchymal tissue compartments, Pitx2 is mostly an endodermal product while Sfrp2 is predominantly expressed in the mesenchymal compartment (**Figure 3B**). In the absence of the LMα5 chain, endodermal expression of Pitx2 is increased while expression of Sfrp2 is noticeable in both compartments.

To examine whether laminin-511 directly influences Wnt signaling, we performed the TOPflash reporter assay using HEK293 cells that do not produce this isoform. As shown in **Figure 3C**, on a laminin-511 substratum TCF-dependent reporter activity is repressed (about 3-fold decrease) in contrast to a laminin-111 coated surface. Laminin-511 dependent inhibition of the TOPflash reporter construct is also observed when cells were transfected with plasmids encoding TCF4 and stabilized β-catenin (not shown). These data suggest that cell adhesion to a laminin-511 substratum blocks Wnt signaling. Next, our goal was to check if adhesion to laminin-511 also negatively regulates Wnt signaling in intestinal cells. Unfortunately, established intestinal cell lines either express LMα5 (personal data, not shown) or are known to exhibit mutations in the Wnt pathway [29]. Therefore, we generated stable *lama5* deficient cells from the non-cancerous m-IC_Cl2_ epithelial cell line using a lentivirus strategy. Two of the five knockdown m-ICc_l2_ cell lines showed about 70% of lama5 inhibition and thus were used for TOPflash activity measurement. As shown in **Figure 3C**, both sh-*lama5* intestinal cell lines seeded on laminin-511 showed a statistically significant inhibition of Wnt activity (3.5-fold decrease) which was in contrast to cells seeded on laminin-111.

### Laminin-511 stimulates expression and activity of the survival factor Akt

Expression of genes belonging to the PI3K/Akt signaling pathway such as Rasgrp2, Pik3cd, Pikc2g, Akt2 is modified in the absence of the LMα5 chain (**Figure 1**). Considering the central role of the serine-threonine protein kinase Akt in cell survival, we analysed the regulation of this enzyme by laminin-511.

**Figure 4:**
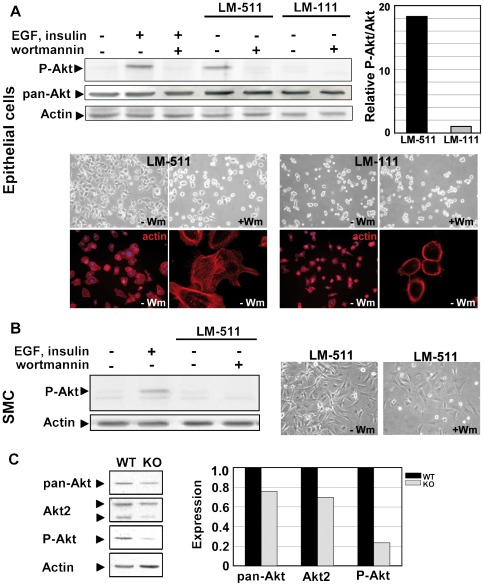
Cell survival and activation of Akt in epithelial cells adhering to laminin-511. (A) m-IC_Cl2_ epithelial cells and (B) embryonic smooth muscle cells (SMC) were plated on uncoated dishes +/− EGF and insulin, and on dishes containing laminin-511 (LM-511) or laminin-111 (LM-111). The PI3K inhibitor wortmannin was added where indicated. Cell lysates were analyzed by western blotting for phosphorylated Akt (P-Akt), Akt (pan-Akt) and actin. Note that activation of Akt is detectable in epithelial cells cultured on laminin-511 but not on laminin-111 (see quantification of the representative gel). No activation occurred in smooth muscle cells in the presence of laminin-511. In parallel, epithelial (A) and smooth muscle cells (B) were photographed by phase contrast microscopy on laminin-511 matrix (LM-511) and on laminin-111 (LM-111) with or without wortmannin (Wm). Cell spreading on laminin-511 (left pictures) versus laminin-111 (right pictures) was confirmed by flattening of the cells and reorganization of the cytoskeleton as probed with TRITC-phalloidin to visualize F-actin. (C) Representative immunoblots showing the expression of Akt (pan-Akt), Akt2 and Phospho-Akt (P-Akt) in E15.5 control (WT) and knockout (KO) intestines and quantification of two independent experiments as ratio between KO (grey bars) and WT (black bars) intestines. Data were normalized using actin.

Activation of Akt was examined in the m-IC_Cl2_ epithelial cell line and in intestinal muscle-derived primary cells that were seeded on laminin-511- or laminin-111-coated surfaces. As shown in **Figure 4A**, while Akt expression is stable whatever the conditions, Akt is phosphorylated in m-IC_Cl2_ cells seeded on laminin-511, but not in cells on laminin-111. To address the role of Akt, we investigated intestinal cell behavior in response to laminin-511 upon inhibition of its upstream regulator PI3K by wortmannin. Addition of this specific inhibitor abolishes Akt phosphorylation in cells cultured on laminin-511 or upon stimulation with growth factors. In contrast to epithelial cells, laminin-511 does not stimulate Akt phosphorylation in muscle cells; yet, Akt can be stimulated by EGF/insulin (**Figure 4B**). Phase contrast microscopy revealed that laminin-511 stimulates spreading of epithelial and muscle cells indistinguishably (**Figure 4A, B**). Spreading of epithelial cells on laminin-511 was visualized by using the actin-binding reagent phalloidin. Cells attached to laminin-111 appeared round while those on laminin-511 were flat and displayed actin in cellular extensions (**Figure 4A**). By confocal microscopy focusing on the basal cell membrane, it was found that cells on laminin-511 were significantly larger and were better spread than on laminin-111 exemplified by extended lamellipodia (**Figure 4A**). Inhibition of Akt with wortmannin abolished spreading of epithelial cells on laminin-511 as evidenced by cell rounding (**Figure 4A**). This is in contrast to muscle cells, which remain spread in the presence of wortmannin (**Figure 4B**). Altogether, our data provide evidence that laminin-511 specifically activates Akt through the PI3K pathway in intestinal epithelial but not in mesenchymal cells.

**Figure 5:**
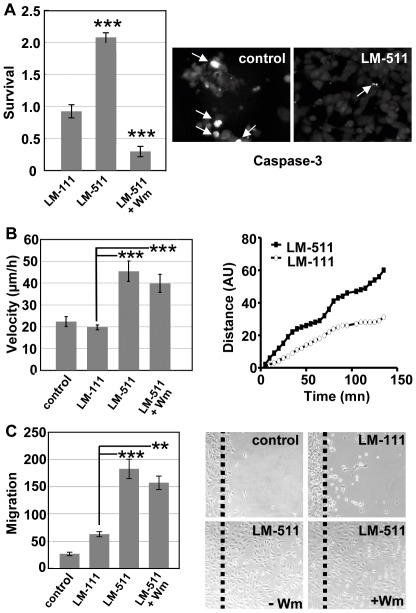
Laminin-511 controls survival and epithelial cell migration. (A) In a survival assay, m-IC_Cl2_ intestinal cells were cultured with H_2_O_2_ on laminin-111 (LM-111), laminin-511 coated-dishes or on laminin-511 (LM-511) with wortmannin (Wm). Survival rates, as ratios normalized to plastic, were determined by a MTS assay. Note a better cell survival rate on laminin-511 as compared to laminin-111, which is abolished upon treatment with Wm (mean +/− SEM, n = 5) (*** p<0.001). Immunofluorescence pictures (right) show more caspase-3-positive cells (arrows) on uncoated dishes (control) as compared to laminin-511. (B) Both migration velocity and cumulative migration distance of cells are significantly enhanced when m-IC_Cl2_ cells are seeded on laminin-511 (LM-511; +/− wortmannin: Wm) versus laminin-111 (LM-111) or uncoated dishes (control) (mean +/− SEM, n = 5) (*** p<0.001). (C) Chemotactic migration of m-IC_Cl2_ cells was visualized by phase contrast microscopy and cell counting on uncoated dishes (control), laminin-111 (LM-111), and laminin-511 (LM-511) in the presence or absence of wortmannin (Wm). Note that laminin-511 stimulated significantly cell migration independently of the PI3K/Akt pathway. In both assays, wortmannin did not affect laminin-511 enhanced migration. The dotted line represents the starting point of migration. Data (n≥5) are given as mean +/− SEM;** p<0.01; *** p<0.001.

Since adhesion is important for survival [30] and laminin-511 supports adhesion of epithelial cells and Akt phosphorylation, we triggered apoptosis by H_2_O_2_ and investigated cell survival in the presence or absence of a laminin-511 substratum and upon treatment with wortmannin by using the MTS assay. We observed that laminin-511 protects cells against H_2_O_2_-induced apoptosis since cell survival is increased statistically by 2-fold as compared to cells seeded on plastic or on laminin-111 (**Figure 5A**). Moreover, the apoptosis protecting effect of laminin-511 is completely abolished by wortmannin (**Figure 5A**). A fraction of control cells (about 30%) exhibited caspase-3 immunoreactivity. This was in contrast to less than 3% of cells on laminin-511 (**Figure 5A, right panel**).

In accordance with the cell culture results, Western blot analysis revealed that total Akt or Akt2 protein expression as well as Akt phosphorylation were lower in LMα5-deficient intestinal tissues in comparison to the control tissue (**Figure 4C**).

### Laminin-511 activates migration of intestinal epithelial cells

Both laminin-511 [16] and PI3K [31] play a role in cell migration, therefore we determined whether laminin-511-specific migration is PI3K dependent. m-IC_Cl2_ epithelial cells were seeded at low density on laminin-511- or laminin-111-coated surfaces and motility was recorded by time-lapse video microscopy. We found that the migration speed is enhanced by laminin-511 as compared to laminin-111 or plastic (**Figure 5B**). Nevertheless, cell trajectories and F-actin cytoskeleton are similar on both laminin substrata (data not shown). But in contrast to cell survival, laminin-511 dependent migration is not PI3K dependent, since wortmannin does not affect random cell migration (**Figure 5B**). To address whether PI3K had an effect on directed migration on laminin-551, m-IC_Cl2_ epithelial cells were plated on this substratum, and migration was initiated upon tilting the dish into a horizontal position. Here also cell migration is significantly increased on laminin-511 in comparison to cells seeded on an uncoated dish or on laminin-111, and again laminin-511 directed migration is not inhibited by wortmannin (**Figure 5C**). Enhanced migration on laminin-511 occurred even in the presence of the DNA synthesis inhibitor mitomycin (not shown) which indicates that the laminin-511 stimulated migration is independent of proliferation. Together, our data show that laminin-511 triggers migration of intestinal epithelial cells in a PI3K independent manner.

### Expression of the LMα5 chain is modified in human intestinal pathologies

**Figure 6:**
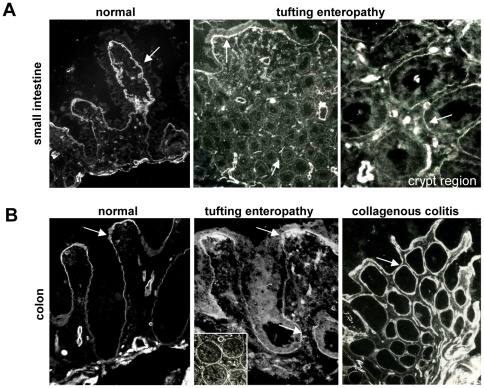
Deregulated LMα5 expression in the intestine of patients with tufting enteropathy and collagenous colitis. LMα5 detection on small intestine (A) and colon (B) from patients with tufting enteropathy and collagenous colitis reveal an abnormal location of this chain as compared to controls. While α5 is detected mostly at the villus compartment in control specimen, it is found also in the crypt region (inset) in tufting enteropathy specimen. In a specimen of collagenous colitis, the staining is stronger all over the crypt-villus axis. Arrows point to the BM region.

In normal human intestine, the LMα5 chain is present in both the subepithelial and the muscle BM [9], [10]. By immunodetection of LMα5, we and others had demonstrated some major modifications of the expression and localization of this chain in pathological intestinal tissue [21], [22]. The normal gut mucosa displays a striking gradient of LMα5 expression along the crypt villus axis with a high expression in the villus and low expression in the crypts [9], [10]. Examination of samples from the small intestine and colon of infants with tufting enteropathy, an epithelial dysplasia, or from collagenous colitis reveals a strong up-regulation of LMα5 in the crypt region (**Figures 6A and B**). Such an altered expression of LMα5 was also noted in the small intestinal mucosa of Crohn's disease ([**21**] and our unpublished data) but not in celiac intestinal mucosa [32].

Together an increase of LMα5 expression in human intestinal tissue with signs of pathologies points to an important role of this chain in intestinal tissue homeostasis.

## Discussion

**Figure 7:**
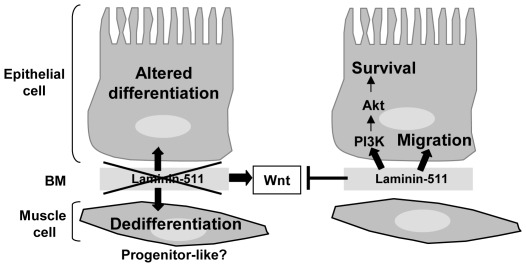
Schematic of the role of laminin-511 in intestinal tissue homeostasis. Laminin-511 is deposited in the intestine by both epithelial and mesenchymal cells [11]. Its absence (KO model or RNA knockdown experiments) or presence (*in vitro* assays) leads to activation or inactivation of Wnt and PI3Kinase signaling pathways promoting multiple cellular responses that impact on survival, migration and differentiation.

The prominent expression of laminin-511 in tissues including the intestine suggests that this laminin isoform plays an important role in tissue homeostasis. Indeed, as shown here and by others [18], [21], [22] uncontrolled overexpression in pathological intestinal tissue or lack of LMα5 leads to severe phenotypes and strongly suggests that LMα5, present in epithelial and muscle BMs, is essential for intestinal tissue morphogenesis. Gene ablation in the mouse reveals that LMα5 is essential since LMα5 deficient mice are early embryonic lethal [12]. Moreover the intestine of these mice is deranged and exhibits in particular a muscle fusion phenotype [18]. To tackle the role of laminin-511 in intestinal homeostasis and cell behavior, we used RNA profiling of embryonic intestinal tissue of *lama*5 knockout mice combined with cell culture experiments. The involvement of some of identified candidate genes has been investigated in endoderm/epithelial and mesoderm/smooth muscle cells isolated by microdissection or using cell culture experiments. Our data provide a mechanistic explanation for the consequences of LMα5 gene deficiency resulting in an aberrant intestinal anatomy. Two major results arise from the present study: loss of the LMα5 chain alters the intestinal gene expression signature and LMα5 is involved in regulating Wnt and PI3K signaling causing probably cell type-specific responses (**Figure 7**).

During intestinal morphogenesis a subset of mesenchymal cells differentiate into smooth muscle cells. This differentiation is accompanied by morphological changes of the cells such as elongation and alignment of mesenchymal cells, and expression of smooth muscle cell markers. In the absence of laminin-511 we observed an altered expression of mesoderm specific genes which indicates an abnormal differentiation and function. In parallel, metabolic enzymes of the intestinal epithelium as well as markers of the brush borders are also downregulated in the absence of *lama5* again suggesting an aberrant differentiation. The fact that LMα5 is required for the establishment and maintenance of the small intestinal epithelium is also supported by the observation that instead of the typical crypt-villus morphology the small intestine of *lama5*-null mice presented a colon-like architecture together with an altered structure of goblet cell granules [6], [19].

Canonical Wnt signaling controls a variety of biological processes including embryonic patterning and colorectal cancer development. In the developing fetal intestine Wnt activity appears around E16 and in postmitotic cells [33]. We found that several Wnt signaling target genes are induced in the absence of *lama5*, amongst them Pitx2 and Msx1 in the endoderm as well as MyoD1 and Hlx in the mesoderm. Activation of Wnt signaling in the absence of *lama5* suggests that Wnt signaling is repressed in a *lama*5 expressing tissue. Indeed our cell culture experiments provide evidence that a laminin-511 substratum represses canonical Wnt signaling, although we did not see nuclear localisation of β-catenin in the *lama5*-deficient intestine (not shown). Yet, activation of this pathway can occur without accumulation of β-catenin in the nucleus as described in the intestine undergoing morphogenesis [34]. Our results are in agreement with the antiparallel expression of LMα5 and Wnt activity in the adult intestine. Indeed LMα5 chain expression depicts a decreasing gradient from the intestinal lumen toward the crypt region which is opposed to Wnt activity [3], [33]. Wnt signaling may potentially modulate muscle differentiation in the *lama5*-deficient intestine. In the mesoderm, the lack of *lama5* leads to disorganization of muscle cells [18] and alters differentiation which could provide an explanation for the muscle fusion phenotype. It is intriguing to note that MyoD and Hlx1 positive muscle cells turned up in the absence of LMα5, suggesting an activation and expansion of myoblast progenitor cells [35], [36]. Moreover in accordance with this concept of dedifferentiation, it was seen that Hlx decreased the activity of promoters of smooth muscle differentiation markers [36].

Although we did not provide evidence of the mechanism by which laminin-511 function to regulate Wnt signaling, one can argue for the involvement of cellular receptors. Indeed, in the laminin-511 deficient intestine we observed an altered expression of matrix adhesion receptors: whereas integrins αv, αM, β4 and the 67 kD laminin receptor are induced, expression of Lutheran is inhibited. It was shown that in the developing kidney canonical Wnt signaling was regulated *in vivo* and *in vitro* by integrin alpha3beta1 and was dependent on the interaction with LM [37]. Furthermore, integrin/Lutheran receptors by themselves might not be the sole actors. As an example, the alpha3beta1 integrin acts in coordination with c-Met (a receptor tyrosine kinase) to regulate the expression of Wnt7b in mouse [37]. In the case of cutaneous development, where the beta1 integrin binding domain of LM-511 is required, a complex loop is implicated between the Shh signaling pathway, PDGF and Wnt signaling [38]

By using cell adhesion assays, we showed that laminin-511 promotes spreading of intestinal epithelial and muscle cells, increases proliferation and migration, and enhances survival of epithelial cells. Enhanced adhesion/proliferation on laminin-511 has already been reported for human colon adenocarcinoma cells, keratinocytes or hematopoietic progenitor cells [39]. Besides that, here we demonstrate that laminin-511 prevents apoptosis via a PI3K-dependent pathway, while it was shown that on fibronectin survival signals are conveyed by the FAK/MEK/ERK pathway [40]. Our data extend published observations made in lung cancer cells [40] by demonstrating an important role of laminin-511 on survival of normal intestinal epithelial cells in a physiological setting. More recently, laminin-511 has been shown to provide an artificial niche that supports the survival of pluripotent human embryonic stem cells allowing their long-term self-renewal [41]. We also observed that intestinal epithelial cells migrate towards laminin-511, but this occurs in a PI3K-independent manner. The described role of laminin-511 in cell migration is in agreement with previously published data [42], [43] and may support its important role in metastasis [44], [45].

What do we learn about the physiological role of *lama5* from these experiments? We demonstrated that cell responses toward a laminin-511 and laminin-111 substratum are clearly distinct. A laminin-511 substratum prevents chemical-induced apoptosis via a PI3K-dependent manner and represses Wnt signaling whereas a laminin-111 substratum does not. Of interest is the fact that these two signaling pathways – PI3K/Akt and Wnt – are interconnected suggesting a potential cross-regulation of transcriptional activity by laminin-511 [46]. Our data also suggest distinct functions of each of the two laminins which is further supported by data from knockout mice. Indeed, SOX2-Cre-mediated knockout of *lama1* in the embryo proper does not interfere with viability [47] or normal morphogenesis of the intestine (Lefebvre *et al*., unpublished data) which is in contrast to the *lama5* deficient mice that die early in embryogenesis [12]. Thus integration of cell responses toward laminin-511 and laminin-111 may be crucial for normal development of the intestine. Moreover, our data suggest that an uncontrolled expression of laminins leads to pathologies of the intestine.

## Materials and Methods

### Biological material and epithelial/mesenchymal intestinal dissociation

Snap-frozen bowel specimens from 6 children displaying tufting enteropathy and from 4 control children were obtained at the hospital Necker-Enfants Malades (Paris, France, Dr O. Goulet) [20], [48]. Colon specimen from a patient with collagenous colitis was obtained at the CHRU Nice (France, Dr A. Rampal).

Embryos of *lama5*-deficient mice [12] were removed by caesarean section. For epithelial/mesenchymal dissociation, embryonic intestine of 13.5-day old mice were treated with a collagenase solution [49]. All experiments were performed in accordance with the INSERM institutional guidelines for animal care (Institutional approval ID: INSERM E67-482-21).

### Generation of cDNA microarray and data analysis

The microarrays used for the transcriptome analysis contained 10,752 murine cDNA clones obtained from five different cDNA bank sources [50], corresponding to 2150 genes. The preparations of fluorescent probes, hybridization step, scanning and quantitative image analysis are detailed in Methods S1. All data is MIAME compliant and the raw data has been deposited in a MIAME compliant database (accession number: GEO GSE31334).

### Semi-quantitative and quantitative RT-PCR

At least 3 paired intestinal control and knockout samples taken from distinct litters were used. Primer sequences are described in **Table S2**. For further information, see Methods S1.

### RNA interference and lentivirus-mediated shRNA interference

RNA interference for *lama5* was performed in embryonic mesenchymal and adult muscle cells with siRNA sequence [51] and negative control siRNA (Eurogentec, Seraing, Belgium, 5′-CAGGACUGCCAGUAGACAdTdT). siRNA were transfected using INTERFERin (Polyplus-transfection, Illkirch, France) as described by the manufacturer. siRNA treated cells were incubated at 37°C for 72 h before RNA extraction, to assess *lama5* and Hlx1 expression by RT-qPCR. Five different MISSION^R^ lentiviral shRNA clones for mouse *lama5* and a non-target shRNA control lentivirus (Sigma-Aldrich, St Louis, MO) were tested in a first round in m-IC_Cl2_ cells. Populations of lentiviral m-IC_Cl2_ infected cells were selected using 0.2 µg/ml puromycin (Invitrogen, Cell culture, France). Efficiency of *lama5* inhibition was determined by RT-qPCR. Then, two stable m-ICc_l2_ – sh-*lama5* (1) and sh-*lama5* (2) – cell lines that inhibit the most lama5 expression were selected for further TOPflash experiments.

### Cell cultures; survival and migration assays

Immortalized mouse intestinal m-IC_Cl2_ cells [52], mesenchymal primary cultures from embryonic intestinal tissue derived from wild-type or LMα5 deficient mice [53] and muscle-derived primary cell cultures were used. Cells were established and cultured as described in the Methods S1 section. Survival and migration assays were done on m-IC_Cl2_ cells as described in Methods S1.

### Akt activity assay

After an overnight serum starvation, m-IC_Cl2_ and muscle-derived primary cells were plated on tissue culture dishes with or without laminin matrix as previously described [39]. Control of Akt activation was performed by adding EGF (0.02 µg/ml; Sigma) and insulin (5 µg/ml; Sigma) to the culture medium. In some experiments, wortmannin, a PI3K inhibitor, was added at a final concentration of 1.5 µM. Cell lysates were obtained as described in Turck *et al* [54]. Akt activity is also determined on homogenized intestinal tissue of LMα5 deficient mice.

### Plasmids, transfection experiments, and TCF/β-catenin reporter assays

The TOPflash reporter vector was used to evaluate activity of Wnt signaling. HEK293 cells (ATCC) and lentiviral m-IC_Cl2_ infected cells were plated into 24-well plates coated or not with laminin-111, Caco-2 derived laminin-511 or recombinant human laminin-511 (BioLamina AB, Sweden). Luciferase assays were performed 24 h later on cell lysates (Dual Luciferase Reporter assay system, Promega; Lumistar luminometer, BMG Labtech, Germany).

### Statistical analysis

Statistical analysis was performed using the one sample t-test (**Figures 2A, 2C** **and** **3B** **; Figure S4**) or the Mann Whitney non parametric test (HEK293 cells in **Figure 3C**) depending on the normality (tested with the Kolmogorov-Smirnov test). The one way Anova test followed by a Tukey's multiple comparison test was used for the other results.

## Supporting Information

Figure S1 **Laminin α5 deficiency leads to a disorganized intestinal morphogenesis.** (A) Laminin-511 is a heterotrimer consisting of a α5, a β1 and a γ1 chain. (B) Views of the proximal portion of the embryonic intestine that reveals excessive folding (arrows) in absence of LMα5 (KO) as compared to controls. (C) Fusion of the external smooth muscle cell layer was sometimes observed as shown here on a semi-thin section stained with Toluidin Blue; sm: smooth muscle. For further details see in Bolcato-Bellemin et al., Dev. Biol. 2003; 260:376–390.
(TIF)

Figure S2 **Laminin α5-specific gene clusters in intestinal tissue.** Selected clusters produced by a hierarchical clustering program are shown on the left. The top clusters follow a pattern of increased expression in laminin α5−/− intestines. In the bottom, clusters of genes are shown whose expression is reduced in knockout intestine (KO) as compared to wild type intestine (WT). Since the severity of the phenotype was heterogenous between samples a similar change in expression in at least 2 of 4 independent experiments with at least a two-fold difference in expression between normal and laminin α5 deficient intestinal tissue was used as criteria. The branch lengths indicate the correlation with which genes were joined, with longer branches indicating a lower correlation. On the right, enlargement of a region with a decreased expression in the knockout and with the listing of the corresponding genes. Each column represents a single experiment, and each row represents a single gene. Increased expression is displayed in red, repressed expression is shown in green and unchanged expression in black, with the relative log2 (ratio) reflecting the intensity of expression. The co-hybridization strategy used in our cDNA microarray experiments controls array variations through data normalization with a common reference, thereby generating data that can be compared between multiple samples. Similar results were obtained from samples derived from independent litters. Note that the controls (WT, L1 to L4) appear as a separate group distinct from the knockout group (KO, L5 to L8).
(TIF)

Figure S3 **Immunodetection of integrin β4 and β1 subunits, and of Lutheran in control and laminin α5 knockout embryonic intestinal and lung tissue.** Basal staining of the β4 subunit was increased concomitant to the lack of laminin α5 chain in the intestine (arrows); at the opposite epithelial Lutheran immunoreactivity was strikingly decreased in both intestine and lung. e, endoderm, m, mesenchyme; s, serosal layer; arrows point to the subepithelial basement membrane region.
(TIF)

Figure S4 **Validation of candidate genes involved in epithelial cell differentiation.** Semi-quantitative RT-PCR experiments were performed on E15.5 control and knockout intestines for genes belonging to the epithelial compartment. Data are presented as fold changes in expression between knockout (grey bars) and wild-type (black bars) (mean +/− SEM, n = 5 to n = 7) (* p<0.05).
(TIF)

Table S1 **Upregulated and downregulated genes in the absence of α5 chain.**
(PDF)

Table S2 **Sequences of the primers used for RT-PCR or RT-qPCR.**
(PDF)

Methods S1 **Supplementary materials and methods.**
(DOC)
