# Supplementary figures and images for: Abnormal Wnt and PI3Kinase Signaling in the Malformed Intestine of lama5 Deficient Mice
Source: PLoS One. 2012 May 30;7(5):e37710. doi: 10.1371/journal.pone.0037710 (PMC3364287; doi:10.1371/journal.pone.0037710)

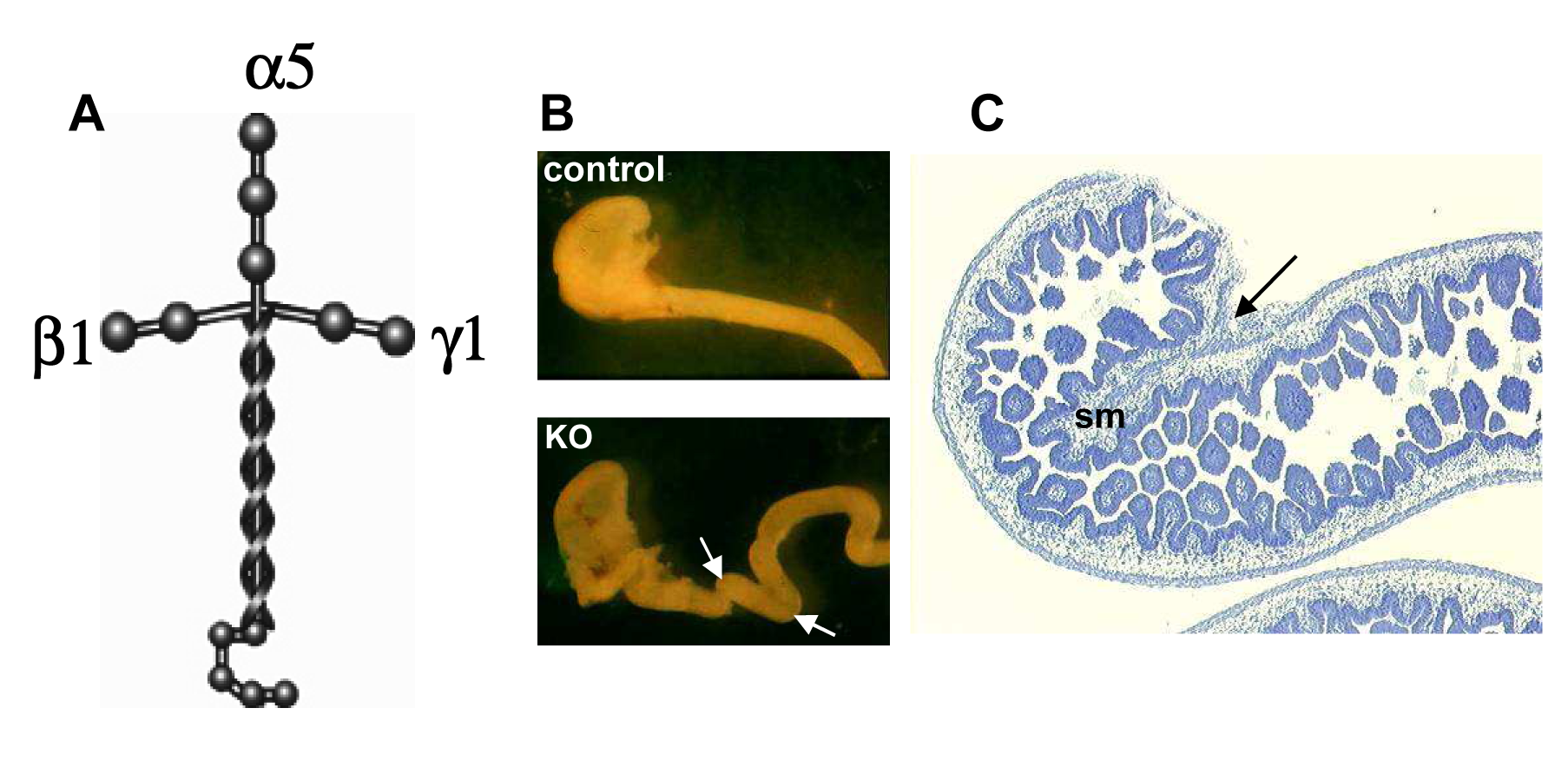

Supplement: Figure S1 — Laminin α5 deficiency leads to a disorganized intestinal morphogenesis. (A) Laminin-511 is a heterotrimer consisting of a α5, a β1 and a γ1 chain. (B) Views of the proximal portion of the embryonic intestine that reveals excessive folding (arrows) in absence of LMα5 (KO) as compared to controls. (C) Fusion of the external smooth muscle cell layer was sometimes observed as shown here on a semi-thin section stained with Toluidin Blue; sm: smooth muscle. For further details see in Bolcato-Bellemin et al., Dev. Biol. 2003; 260:376–390. (TIF) [file pone.0037710.s001.tif]

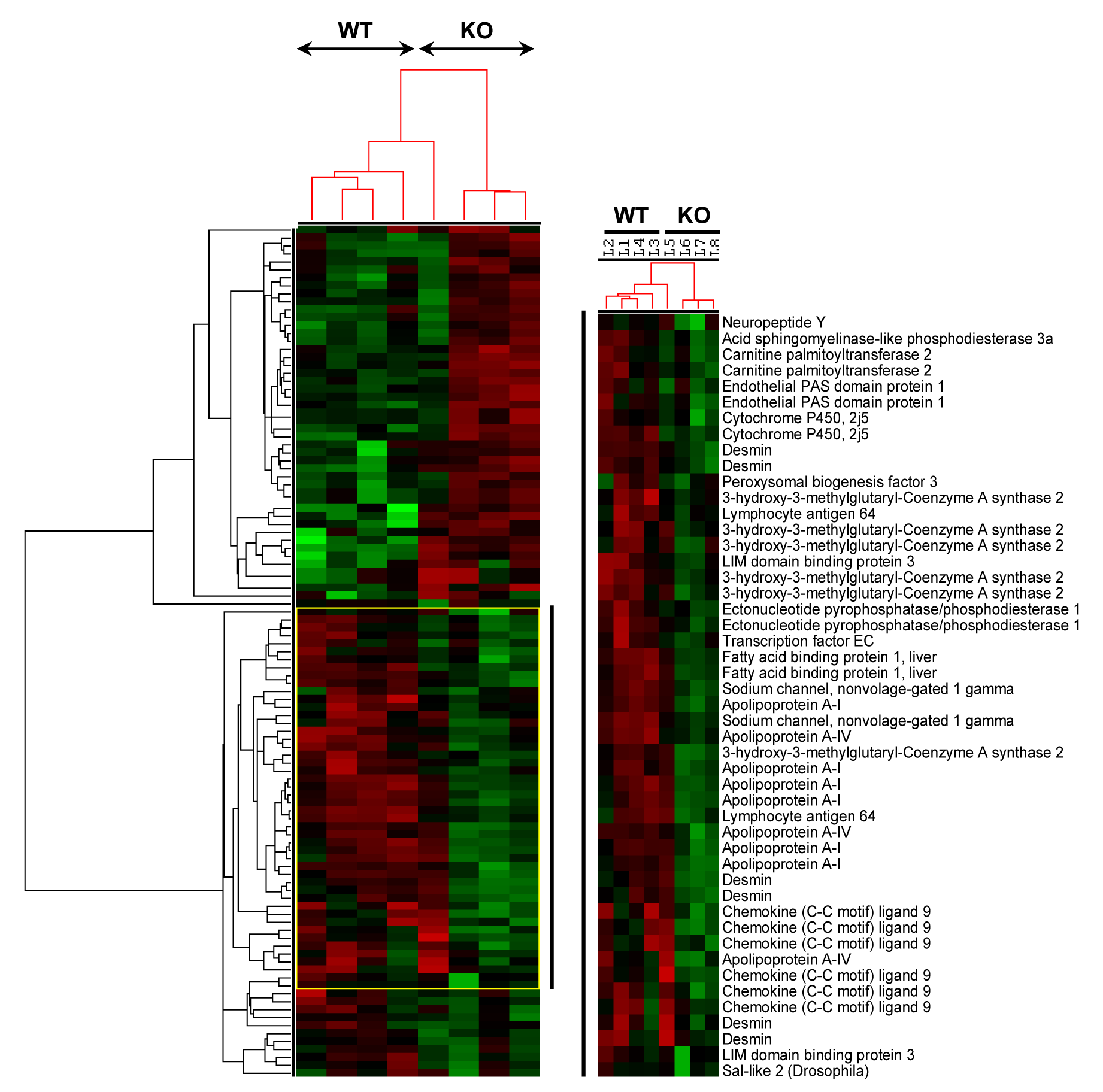

Supplement: Figure S2 — Laminin α5-specific gene clusters in intestinal tissue. Selected clusters produced by a hierarchical clustering program are shown on the left. The top clusters follow a pattern of increased expression in laminin α5−/− intestines. In the bottom, clusters of genes are shown whose expression is reduced in knockout intestine (KO) as compared to wild type intestine (WT). Since the severity of the phenotype was heterogenous between samples a similar change in expression in at least 2 of 4 independent experiments with at least a two-fold difference in expression between normal and laminin α5 deficient intestinal tissue was used as criteria. The branch lengths indicate the correlation with which genes were joined, with longer branches indicating a lower correlation. On the right, enlargement of a region with a decreased expression in the knockout and with the listing of the corresponding genes. Each column represents a single experiment, and each row represents a single gene. Increased expression is displayed in red, repressed expression is shown in green and unchanged expression in black, with the relative log2 (ratio) reflecting the intensity of expression. The co-hybridization strategy used in our cDNA microarray experiments controls array variations through data normalization with a common reference, thereby generating data that can be compared between multiple samples. Similar results were obtained from samples derived from independent litters. Note that the controls (WT, L1 to L4) appear as a separate group distinct from the knockout group (KO, L5 to L8). (TIF) [file pone.0037710.s002.tif]

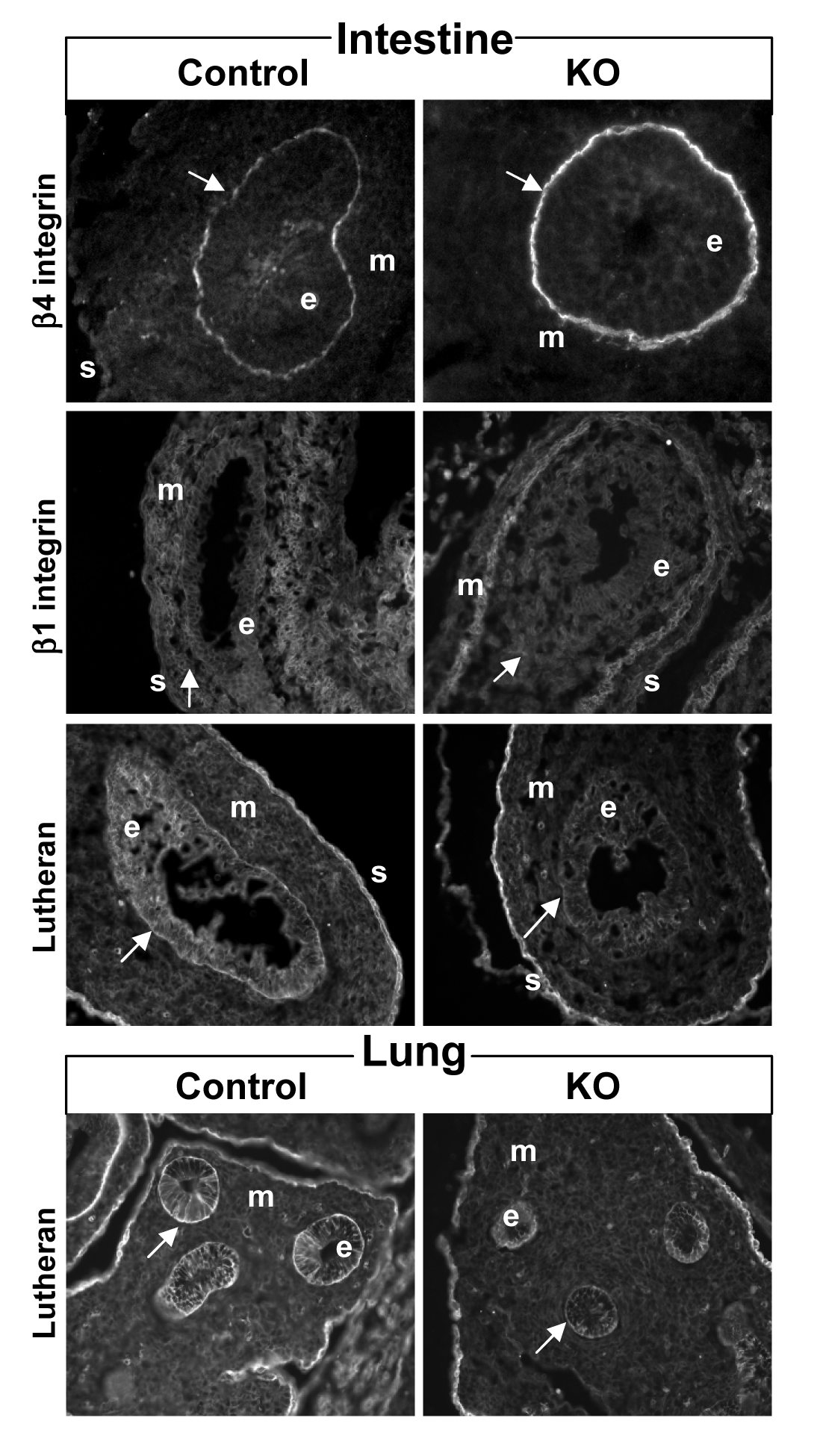

Supplement: Figure S3 — Immunodetection of integrin β4 and β1 subunits, and of Lutheran in control and laminin α5 knockout embryonic intestinal and lung tissue. Basal staining of the β4 subunit was increased concomitant to the lack of laminin α5 chain in the intestine (arrows); at the opposite epithelial Lutheran immunoreactivity was strikingly decreased in both intestine and lung. e, endoderm, m, mesenchyme; s, serosal layer; arrows point to the subepithelial basement membrane region. (TIF) [file pone.0037710.s003.tif]

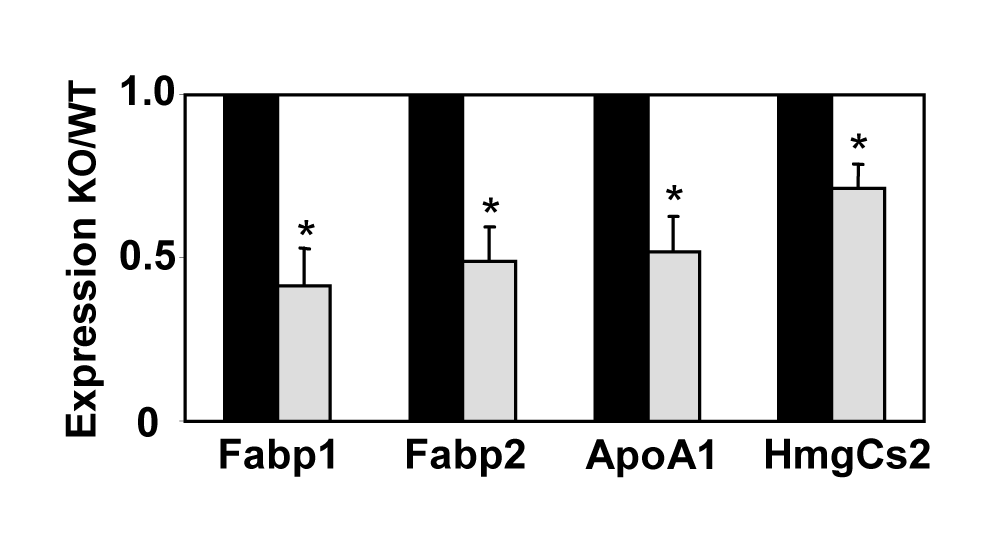

Supplement: Figure S4 — Validation of candidate genes involved in epithelial cell differentiation. Semi-quantitative RT-PCR experiments were performed on E15.5 control and knockout intestines for genes belonging to the epithelial compartment. Data are presented as fold changes in expression between knockout (grey bars) and wild-type (black bars) (mean +/− SEM, n = 5 to n = 7) (* p<0.05). (TIF) [file pone.0037710.s004.tif]
