# Supplementary material for: Abnormal Wnt and PI3Kinase Signaling in the Malformed Intestine of lama5 Deficient Mice
Source: PLoS One. 2012 May 30;7(5):e37710. doi: 10.1371/journal.pone.0037710 (PMC3364287; doi:10.1371/journal.pone.0037710)
Supplement: Table S1 — Upregulated and downregulated genes in the absence of α5 chain. (PDF) [file pone.0037710.s005.pdf]

Table S1

Upregulated genes in the absence of α5 chain

Gene	Accession number	
Tnfsf9	Mm.41171	tumor necrosis factor (ligand) superfamily, member 9"
Hlx	Mm.1347	H2.0-like homeo box gene"
Kcnn4	Mm.9911	potassium intermediate/small conductance calcium-activated channel, subfamily N, member 4"
Deaf1	Mm.28392	deformed epidermal autoregulatory factor 1 (Drosophila)"
Ccnb1-rs1	Mm.258385	cyclin B1, related sequence 1"
Sin3b	Mm.2137	transcriptional regulator, SIN3B (yeast)"
Cd34	Mm.29798	CD34 antigen"
Clta	Mm.198817	clathrin, light polypeptide (Lca)"
Rara	Mm.103336	retinoic acid receptor, alpha"
Bcl2l	Mm.3882	Bcl2-like"
Mapk14	Mm.4437	mitogen activated protein kinase 14"
Mad3	Mm.20350	Max dimerization protein 3"
Klf4	Mm.4325	Kruppel-like factor 4 (gut)"
Kifc1	Mm.197684	kinesin family member C1"
	Mm.40966	Mus musculus adult male corpora quadrigemina cDNA, RIKEN full-length enriched library, clone:B230309J24 product:hypothetical Zinc finger, C2H2 type containing protein, full insert sequence."
Cryaa	Mm.1228	crystallin, alpha A"
Heyl	Mm.103615	hairy/enhancer-of-split related with YRPW motif-like"
Csf1	Mm.795	colony stimulating factor 1 (macrophage)"
Prkcq	Mm.2921	protein kinase C, theta"
Pitx2	Mm.1385	paired-like homeodomain transcription factor 2"
Lhx1	Mm.4965	LIM homeobox protein 1"
Ppp2r1a	Mm.1034	protein phosphatase 2 (formerly 2A), regulatory subunit A (PR 65), alpha isoform"
Hmgb3	Mm.340	high mobility group box 3"
Klf1	Mm.4847	Kruppel-like factor 1 (erythroid)"
Elavl3	Mm.3477	ELAV (embryonic lethal, abnormal vision, Drosophila)-like 3 (Hu antigen C)"
Rpa2	Mm.2870	replication protein A2"
Sema4d	Mm.33903	sema domain, immunoglobulin domain (Ig), transmembrane domain (TM) and short cytoplasmic domain, (semaphorin) 4D"
Sema4a	Mm.22061	sema domain, immunoglobulin domain (Ig), transmembrane domain (TM) and short cytoplasmic domain, (semaphorin) 4A"
Mapk9	Mm.68933	mitogen activated protein kinase 9"
Cul3	Mm.12665	cullin 3"
Flt3	Mm.194	FMS-like tyrosine kinase 3"
Pscd2	Mm.19097	pleckstrin homology, Sec7 and coiled/coil domains 2"
Rpo2tc1	Mm.41746	RNA polymerase II transcriptional coactivator"
Hba-x	Mm.141758	hemoglobin X, alpha-like embryonic chain in Hba complex"
Ntrk2	Mm.3993	neurotrophic tyrosine kinase, receptor, type 2"
Tal1	Mm.3102	T-cell acute lymphocytic leukemia 1"
H2-Eb1	Mm.22564	histocompatibility 2, class II antigen E beta"
Scn10a	Mm.42141	sodium channel, voltage-gated, type X, alpha polypeptide"
Syt4	Mm.233846	synaptotagmin 4"
Pik3cd	Mm.153755	phosphatidylinositol 3-kinase catalytic delta polypeptide"
Bcl10	Mm.28782	B-cell leukemia/lymphoma 10"
Hmgb1	Mm.16421	high mobility group box 1"
Il11ra2	Mm.193451	interleukin 11 receptor, alpha chain 2"

Eif1a	Mm.143141	eukaryotic translation initiation factor 1A"
Alox12e	Mm.1122	arachidonate lipoxygenase, epidermal"
Arf5	Mm.4996	ADP-ribosylation factor 5"
H2-K	Mm.16771	histocompatibility 2, K region"
Meox2	Mm.153716	mesenchyme homeobox 2"
Top3a	Mm.10708	topoisomerase (DNA) III alpha"
Sypl	Mm.45146	synaptophysin-like protein"
Gprk6	Mm.10193	G protein-coupled receptor kinase 6"
Tcrb-V8.3	Mm.138641	T-cell receptor beta, variable 8.3"
Il4	Mm.371	interleukin 4"
Uox	Mm.10865	urate oxidase"
inosine moP		
Apoa2	Mm.43677	apolipoprotein A-II"
Il17r	Mm.4481	interleukin 17 receptor"
Madh2	Mm.2334	MAD homolog 2 (Drosophila)"
Cnn3	Mm.22171	calponin 3, acidic"
Trp63	Mm.20894	transformation related protein 63"
Apoe	Mm.156335	apolipoprotein E"
Gnb2-rs1	Mm.5305	guanine nucleotide binding protein, beta 2, related sequence 1"
Gata3	Mm.606	GATA binding protein 3"
Tnfrsf9	Mm.198677	tumor necrosis factor receptor superfamily, member 9"
Mor1	Mm.21743	malate dehydrogenase, mitochondrial"
PHC ETA		
Il13ra1	Mm.24208	interleukin 13 receptor, alpha 1"
Igfbp3	Mm.29254	insulin-like growth factor binding protein 3"
Igf2bp3	Mm.18496	insulin-like growth factor 2, binding protein 3"
Hspa5	Mm.918	heat shock 70kD protein 5 (glucose-regulated protein)"
ACTG1 (Hs)		
Arha2	Mm.757	ras homolog gene family, member A2"
Eif2b	Mm.140365	eukaryotic translation initiation factor 2B"
1110002L01Rik	Mm.36793	RIKEN cDNA 1110002L01 gene"
Pcsk5	Mm.3401	proprotein convertase subtilisin/kexin type 5"
Hspd1	Mm.1777	heat shock protein 1 (chaperonin)"
Rad9	Mm.193035	RAD9 homolog (S. pombe)"
Prrx2	Mm.1802	paired related homeobox 2"
Rgs19	Mm.20156	regulator of G-protein signaling 19"
Mg29	Mm.20942	mitsugumin 29"
Mapt	Mm.1287	microtubule-associated protein tau"
Nbl1	Mm.9404	neuroblastoma, suppression of tumorigenicity 1"
Cbx5	Mm.28003	chromobox homolog 5 (Drosophila HP1a)"
2900026H06Rik	Mm.151483	RIKEN cDNA 2900026H06 gene"
Apoc1	Mm.182440	apolipoprotein C-I"
Cxcl12	Mm.465	chemokine (C-X-C motif) ligand 12"
E2f5	Mm.379	E2F transcription factor 5"
Sox4	Mm.18789	SRY-box containing gene 4"
Tubb5	Mm.1703	tubulin, beta 5"
Dlx5	Mm.4873	distal-less homeobox 5"
Itpr3	Mm.26945	inositol 1,4,5-triphosphate receptor 3"
Kif1a	Mm.4762	kinesin family member 1A"
	Mm.218722	Mus musculus, Similar to maternal G10 transcript, clone IMAGE:3594956, mRNA"
Ebf1	Mm.4366	early B-cell factor 1"
	Mm.23130	ESTs"
Map2k3	Mm.18494	mitogen activated protein kinase kinase 3"
2610029D06Rik	Mm.41554	RIKEN cDNA 2610029D06 gene"
MGC18735	Mm.100403	hypothetical protein MGC18735"
Prim1	Mm.2903	DNA primase, p49 subunit"
Dermo1	Mm.9474	dermis expressed 1"
Pex6	Mm.41268	peroxisomal biogenesis factor 6"

Smarca2	Mm.181163	SWI/SNF related, matrix associated, actin dependent regulator of chromatin, subfamily a, member 2"
Gnai2	Mm.196464	guanine nucleotide binding protein, alpha inhibiting 2"
Hbb-y	Mm.35830	hemoglobin Y, beta-like embryonic chain"
Prkdc	Mm.71	protein kinase, DNA activated, catalytic polypeptide"
Mre11a	Mm.25544	meiotic recombination 11 homolog A (S. cerevisiae)"
Ldb3	Mm.29733	LIM domain binding 3"
Epha1	Mm.133330	Eph receptor A1"
Rab33a	Mm.2015	RAB33A, member of RAS oncogene family"
Mknk1	Mm.206467	MAP kinase-interacting serine/threonine kinase 1"
Rpo2tc1	Mm.41746	RNA polymerase II transcriptional coactivator"
Ccl22	Mm.12895	chemokine (C-C motif) ligand 22"
Nr4a3	Mm.101224	nuclear receptor subfamily 4, group A, member 3"
Dlk1	Mm.157069	delta-like 1 homolog (Drosophila)"
Rasgrp2	Mm.20884	RAS, guanyl releasing protein 2"
Adora2a	Mm.153916	adenosine A2a receptor"
Rrm1	Mm.656	ribonucleotide reductase M1"
Tnfrsf10b	Mm.193430	tumor necrosis factor receptor superfamily, member 10b"
Chek1	Mm.16753	checkpoint kinase 1 homolog (S. pombe)"
Ltb	Mm.1715	lymphotoxin B"
Wars	Mm.38433	tryptophanyl-tRNA synthetase"
Dvl1	Mm.3400	dishevelled, dsh homolog 1 (Drosophila)"
Gata2	Mm.1391	GATA binding protein 2"
Tdo2	Mm.21545	tryptophan 2,3-dioxygenase"
Kcnk2	Mm.31570	potassium channel, subfamily K, member 2"
Sfrp2	Mm.19155	secreted frizzled-related sequence protein 2"
Casp14	Mm.20940	caspase 14"
Itgb4	Mm.21117	integrin beta 4"
Fgf2	Mm.57094	fibroblast growth factor 2"
Ifngr	Mm.549	interferon gamma receptor"
Hsd3b4	Mm.14309	hydroxysteroid dehydrogenase-4, delta<5>-3-beta"
Ii	Mm.258212	Ia-associated invariant chain"
Pfkfb2	Mm.257993	6-phosphofructo-2-kinase/fructose-2,6-biphosphatase 2"
Nfkb1	Mm.3420	nuclear factor of kappa light chain gene enhancer in B-cells 1, p105"
Pte2b-pending	Mm.219001	peroxisomal acyl-CoA thioesterase 2B"
INSULINE		
Bcar1	Mm.3758	breast cancer anti-estrogen resistance 1"
Itgav	Mm.4427	integrin alpha V"
Abcc5	Mm.20845	ATP-binding cassette, sub-family C (CFTR/MRP), member 5"
Cbl	Mm.246818	Casitas B-lineage lymphoma"
Csf2ra	Mm.156264	colony stimulating factor 2 receptor, alpha, low-affinity (granulocyte-macrophage)"
Slc4a3	Mm.5053	solute carrier family 4 (anion exchanger), member 3"
NLSlacZ		
Pik3c2g	Mm.10301	phosphatidylinositol 3-kinase, C2 domain containing, gamma polypeptide"
CystProt (plant)		
IFNGR2		
Maff	Mm.86646	v-maf musculoaponeurotic fibrosarcoma oncogene family, protein F (avian)"
Gla	Mm.1114	galactosidase, alpha"
Trp53	Mm.222	transformation related protein 53"
Erh	Mm.21952	enhancer of rudimentary homolog (Drosophila)"
Msc	Mm.5820	musculin"
Abl1	Mm.1318	v-abl Abelson murine leukemia oncogene 1"
Csrp2	Mm.2020	cysteine-rich protein 2"
Ywhah	Mm.3308	tyrosine 3-monooxygenase/tryptophan 5-monooxygenase activation protein, eta polypeptide"
LOC230904	Mm.23290	hypothetical protein LOC230904"
Gng5	Mm.140804	guanine nucleotide binding protein (G protein), gamma 5 subunit"

Ldb1	Mm.4524	LIM domain binding 1"
Prkar1b	Mm.9334	protein kinase, cAMP dependent regulatory, type I beta"
Itgam	Mm.4967	integrin alpha M"
Rgs5	Mm.20954	regulator of G-protein signaling 5"
Runx2	Mm.4509	runt related transcription factor 2"
Tlr4	Mm.85343	toll-like receptor 4"
Mapkapk2	Mm.29725	MAP kinase-activated protein kinase 2"
Msx1	Mm.870	homeo box, msh-like 1"
Clock	Mm.3552	circadian locomoter output cycles kaput"
Ppy	Mm.1269	pancreatic polypeptide"
Per2	Mm.8471	period homolog 2 (Drosophila)"
Lig3	Mm.1411	ligase III, DNA, ATP-dependent"
Pak3	Mm.3392	p21 (CDKN1A)-activated kinase 3"
Actb	Mm.297	actin, beta, cytoplasmic"
Ppp1r3c	Mm.24724	protein phosphatase 1, regulatory (inhibitor) subunit 3C"
Mif	Mm.2326	macrophage migration inhibitory factor"
Prkag1	Mm.6670	protein kinase, AMP-activated, gamma 1 non-catalytic subunit"
Nr4a1	Mm.119	nuclear receptor subfamily 4, group A, member 1"
Myod1	Mm.1526	myogenic differentiation 1"
Mbp	Mm.2992	myelin basic protein"
Fzd2	Mm.36416	frizzled homolog 2 (Drosophila)"
Slc2a1	Mm.21002	solute carrier family 2 (facilitated glucose transporter), member 1"
Eng	Mm.4851	endoglin"
Ubb	Mm.235	ubiquitin B"
JAK 3		
Lamr1	Mm.4071	laminin receptor 1 (ribosomal protein SA)"
Il12a	Mm.103783	interleukin 12a"
Sc5d	Mm.13081	sterol-C5-desaturase (fungal ERG3, delta-5-desaturase) homolog (S. cerevisae)"
Eef1a1	Mm.196614	eukaryotic translation elongation factor 1 alpha 1"
TGF BETA 1		
Il2rb		
Nfkbie	Mm.57043	nuclear factor of kappa light polypeptide gene enhancer in B-cells inhibitor, epsilon"
AI195443	Mm.157103	expressed sequence AI195443"

Downregulated genes in the absence of α5 chain

Gene	Accession number	
Rfxank	Mm.161167	regulatory factor X-associated ankyrin-containing protein"
Hmgn1	Mm.2756	high mobility group nucleosomal binding domain 1"
8030489C12Rik	Mm.128834	RIKEN cDNA 8030489C12 gene"
Nkx2-2	Mm.4701	NK2 transcription factor related, locus 2 (Drosophila)"
Ywhaz	Mm.3360	tyrosine 3-monooxygenase/tryptophan 5-monooxygenase activation protein, zeta polypeptide"
Ccl9	Mm.2271	chemokine (C-C motif) ligand 9"
Cryaa	Mm.1228	crystallin, alpha A"
Csrp2	Mm.2020	cysteine-rich protein 2"
Amacr	Mm.2787	alpha-methylacyl-CoA racemase"
Es2el	Mm.10094	expressed sequence 2 embryonic lethal"
Rnf4	Mm.21281	ring finger protein 4"
Ldb3	Mm.29733	LIM domain binding 3"
Ldb3	Mm.29733	LIM domain binding 3"
Dad1	Mm.2547	defender against cell death 1"
Mapk1	Mm.196581	mitogen activated protein kinase 1"
Limk2	Mm.42927	LIM motif-containing protein kinase 2"
Np15	Mm.30084	nuclear protein 15.6"

A630031M04Rik	Mm.227621	RIKEN cDNA A630031M04 gene"
Slc8a1	Mm.4211	solute carrier family 8 (sodium/calcium exchanger), member 1"
4833412C19Rik	Mm.60219	RIKEN cDNA 4833412C19 gene"
Sncg	Mm.22231	synuclein, gamma"
Rnf12	Mm.44069	ring finger protein 12"
Nr1i3	Mm.3077	nuclear receptor subfamily 1, group I, member 3"
Chk	Mm.5262	choline kinase"
Ranbp2	Mm.142730	RAN binding protein 2"
Tcl1b1	Mm.27538	T-cell leukemia/lymphoma 1B, 1"
Pparbp	Mm.12926	peroxisome proliferator activated receptor binding protein"
Ctsc	Mm.684	cathepsin C"
Prss25	Mm.21880	protease, serine, 25"
Pitpnb	Mm.200516	phosphotidylinositol transfer protein, beta"
Pitpn	Mm.3128	phosphatidylinositol transfer protein"
Hsp105	Mm.34828	heat shock protein"
Prkag1	Mm.6670	protein kinase, AMP-activated, gamma 1 non-catalytic subunit"
Csf1	Mm.795	colony stimulating factor 1 (macrophage)"
Ndr1	Mm.4063	N-myc downstream regulated 1"
Mafb	Mm.67919	v-maf musculoaponeurotic fibrosarcoma oncogene family, protein B (avian)"
Klf13	Mm.41170	Kruppel-like factor 13"
4833412C19Rik	Mm.60219	RIKEN cDNA 4833412C19 gene"
STAT5a		
Msc	Mm.5820	musculin"
Dvl2	Mm.5114	dishevelled 2, dsh homolog (Drosophila)"
Csnk2a1-rs4	Mm.23692	casein kinase II, alpha 1 related sequence 4"
Hspa5	Mm.918	heat shock 70kD protein 5 (glucose-regulated protein)"
Wnt10b	Mm.4709	wingless related MMTV integration site 10b"
Tuba4	Mm.1155	tubulin, alpha 4"
Hoxa10	Mm.5	homeo box A10"
Lamp2	Mm.486	lysosomal membrane glycoprotein 2"
Hspa1a	Mm.196559	heat shock protein 1A"
Edg3	Mm.136736	endothelial differentiation, sphingolipid G-protein-coupled receptor, 3"
Fhl1	Mm.3126	four and a half LIM domains 1"
Cckar	Mm.3521	cholecystokinin A receptor"
Gna13	Mm.193925	guanine nucleotide binding protein, alpha 13"
Edg2	Mm.4772	endothelial differentiation, lysophosphatidic acid G-protein-coupled receptor, 2"
Ppp1cc	Mm.7793	protein phosphatase 1, catalytic subunit, gamma isoform"
Pex3	Mm.41300	peroxisomal biogenesis factor 3"
EST	Mm.33148	ESTs, Weakly similar to I48668 zinc finger protein 51 - mouse [M.musculus]"
Pps	Mm.1458	putative phosphatase"
Crat	Mm.20396	carnitine acetyltransferase"
Nfe2l2	Mm.1025	nuclear, factor, erythroid derived 2, like 2"
Sc5d	Mm.13081	sterol-C5-desaturase (fungal ERG3, delta-5-desaturase) homolog (S. cerevisae)"
Nsg1	Mm.7414	neuron specific gene family member 1"
Map2k4	Mm.27491	mitogen activated protein kinase kinase 4"
Prdx2	Mm.42948	peroxiredoxin 2"
Cd79a	Mm.1355	CD79A antigen (immunoglobulin-associated alpha)"
Prim2	Mm.27705	DNA primase, p58 subunit"
Lta4h	Mm.533	leukotriene A4 hydrolase"
Cryaa	Mm.1228	crystallin, alpha A"
Calm1	Mm.34246	calmodulin 1"
Gjb1	Mm.21198	gap junction membrane channel protein beta 1"
Pld2	Mm.2538	phospholipase D2"
Axin	Mm.23684	axin"
Mte1-pending	Mm.45431	mitochondrial acyl-CoA thioesterase 1"

Atp9b	Mm.104687	ATPas, class II, type 9B"
Faah	Mm.2915	fatty acid amide hydrolase"
Calm3	Mm.1147	calmodulin 3"
Akt2	Mm.177194	thymoma viral proto-oncogene 2"
Crmp1	Mm.22695	collapsin response mediator protein 1"
Asml3a-pending	Mm.2379	acid sphingomyelinase-like phosphodiesterase 3a"
Cxcl12	Mm.465	chemokine (C-X-C motif) ligand 12"
Mbtps1	Mm.29791	membrane-bound transcription factor protease, site 1"
Cpt2	Mm.29499	carnitine palmitoyltransferase 2"
Icam1	Mm.25455	intercellular adhesion molecule"
Rgs7	Mm.7956	regulator of G protein signaling 7"
Cap1	Mm.8687	adenylyl cyclase-associated CAP protein homolog 1 (S. cerevisiae, S. pombe)"
Homer2	Mm.228	homer homolog 2 (Drosophila)"
Zfp35	Mm.3297	zinc finger protein 35"
Ap1m2	Mm.22239	adaptor protein complex AP-1, mu 2 subunit"
Kif5b	Mm.3380	kinesin family member 5B"
Pde9a	Mm.10812	phosphodiesterase 9A"
Dpp4	Mm.1151	dipeptidylpeptidase 4"
H2-K	Mm.16771	histocompatibility 2, K region"
Hmgn2	Mm.911	high mobility group nucleosomal binding domain 2"
Pnoc	Mm.16347	prepronociceptin"
Stk2	Mm.7693	serine/threonine kinase 2"
Sptlc1	Mm.240336	serine palmitoyltransferase, long chain base subunit 1"
Calb1	Mm.354	calbindin-28K"
Degs	Mm.29648	degenerative spermatocyte homolog (Drosophila)"
Slc11a2	Mm.1304	solute carrier family 11 (proton-coupled divalent metal ion transporters), member 2"
Scp2	Mm.1779	sterol carrier protein 2, liver"
Kcnn4	Mm.9911	potassium intermediate/small conductance calcium-activated channel, subfamily N, member 4"
Ly6a	Mm.8180	lymphocyte antigen 6 complex, locus A"
Rbbp7	Mm.1603	retinoblastoma binding protein 7"
Alas2	Mm.140509	aminolevulinic acid synthase 2, erythroid"
Stk11	Mm.29947	serine/threonine kinase 11"
Rrm1	Mm.656	ribonucleotide reductase M1"
Ddx5	Mm.19101	DEAD/H (Asp-Glu-Ala-Asp/His) box polypeptide 5"
Gata3	Mm.606	GATA binding protein 3"
Mad1l1	Mm.27250	mitotic arrest deficient 1-like 1"
Rab3d	Mm.29968	RAB3D, member RAS oncogene family"
Epas1	Mm.1415	endothelial PAS domain protein 1"
Cd24a	Mm.6417	CD24a antigen"
Rgl2	Mm.43777	ral guanine nucleotide dissociation stimulator,-like 2"
Anpep	Mm.4487	alanyl (membrane) aminopeptidase"
D18Ertd240e	Mm.142498	DNA segment, Chr 18, ERATO Doi 240, expressed"
Stk11	Mm.29947	serine/threonine kinase 11"
Hmgcs2	Mm.10633	3-hydroxy-3-methylglutaryl-Coenzyme A synthase 2"
	Mm.27563	Mus musculus 16 days neonate cerebellum cDNA, RIKEN full-length enriched library, clone:9630047E15 product:unknown EST, full insert sequence."
Rxra	Mm.3470	retinoid X receptor alpha"
Pdlim1	Mm.5567	PDZ and LIM domain 1 (elfin)"
Ccna2	Mm.4189	cyclin A2"
Bcl2l2	Mm.6967	Bcl2-like 2"
Dp1	Mm.21251	deleted in polyposis 1"
Bmp1	Mm.27757	bone morphogenetic protein 1"
Sla	Mm.7601	src-like adaptor"
Stk25	Mm.28761	serine/threonine kinase 25 (yeast)"
Cetn2	Mm.24643	centrin 2"
Rab1	Mm.14530	RAB1, member RAS oncogene family"

5830471N16Rik	Mm.258520	RIKEN cDNA 5830471N16 gene"
Casp14	Mm.20940	caspase 14"
Casp7	Mm.35687	caspase 7"
Npy	Mm.154796	neuropeptide Y"
Fabp1	Mm.22126	fatty acid binding protein 1, liver"
Fabp2	Mm.28398	fatty acid binding protein 2, intestinal"
Apoa4	Mm.4533	apolipoprotein A-IV"
Scnn1g	Mm.35247	sodium channel, nonvoltage-gated 1 gamma"
Apoa1	Mm.26743	apolipoprotein A-I"
Hmgcs2	Mm.10633	3-hydroxy-3-methylglutaryl-Coenzyme A synthase 2"
Enpp1	Mm.27254	ectonucleotide pyrophosphatase/phosphodiesterase 1"
Prlr	Mm.2752	prolactin receptor"
Ddx26	Mm.4173	DEAD/H (Asp-Glu-Ala-Asp/His) box polypeptide 26"
Scp2	Mm.1779	sterol carrier protein 2, liver"
Tfdp1	Mm.925	transcription factor Dp 1"
Sox6	Mm.4656	SRY-box containing gene 6"
Cd44	Mm.24138	CD44 antigen"
Apoa1	Mm.26743	apolipoprotein A-I"
Hspa4	Mm.1032	heat shock protein 4"
Cyp2j5	Mm.12838	cytochrome P450, 2j5"
Facl2	Mm.28962	fatty acid Coenzyme A ligase, long chain 2"
Elavl3	Mm.3477	ELAV (embryonic lethal, abnormal vision, Drosophila)-like 3 (Hu antigen C)"
Sall2	Mm.39487	sal-like 2 (Drosophila)"
Ldb3	Mm.29733	LIM domain binding 3"
Ccl9	Mm.2271	chemokine (C-C motif) ligand 9"
Kitl	Mm.4235	kit ligand"
Cap1	Mm.8687	adenylyl cyclase-associated CAP protein homolog 1 (S. cerevisiae, S. pombe)"
Des	Mm.6712	desmin"
Apoc2	Mm.28394	apolipoprotein C-II"
Tcfec	Mm.36217	transcription factor EC"
Mep1a	Mm.5346	meprin 1 alpha"
Klf3	Mm.28787	Kruppel-like factor 3 (basic)"
Ccl25	Mm.7275	chemokine (C-C motif) ligand 25"
Ly64	Mm.3177	lymphocyte antigen 64"
Acadvl	Mm.18630	acyl-Coenzyme A dehydrogenase, very long chain"
Acadl	Mm.2445	acetyl-Coenzyme A dehydrogenase, long-chain"
Fabp2	Mm.28398	fatty acid binding protein 2, intestinal"
Pdcd8	Mm.30050	programmed cell death 8"
Pdcd2	Mm.323	programmed cell death 2"
Cat	Mm.4215	catalase"
Gnat1	Mm.69061	guanine nucleotide binding protein, alpha transducing 1"
Gpld1	Mm.2779	glycosylphosphatidylinositol specific phospholipase D1"
