# Supplementary material for: Abnormal Wnt and PI3Kinase Signaling in the Malformed Intestine of lama5 Deficient Mice
Source: PLoS One. 2012 May 30;7(5):e37710. doi: 10.1371/journal.pone.0037710 (PMC3364287; doi:10.1371/journal.pone.0037710)
Supplement: Table S2 — Sequences of the primers used for RT-PCR or RT-qPCR. (PDF) [file pone.0037710.s006.pdf]

Gene	Sequences (5' to 3')
36B4	ATG TGA AGT CAC TGT GCC AG
	GTG TAA TCC CTC TCC ACA GA
ApoAI	CAC GTA TGG CAG CAA GAT GAA CC
	TTC TGG AAT TCG TCC AGG TAG
Axin1	GA CTT GGA ACT CTC CGA GAC AGA
	AGT GCA CAG TAG TGA TGG TGT GC
CCKAR	TGCTCAAGGATTTCATCTTCG
	ACCTTCAAAGCATGGGACTTT
Desmin	AGGAGGAGATCCGACACCTAA
	CTCGGAAGTTGAGAGCAGACA
Dvl1	CGA CCT GTG CAG TAA CCT CGC A
	CCA CTT GGT ACT GTG TGG TCT GA
Dvl2	GCT TAC CCT GGC TCC TCA TCT AT
	CCC ACT GGA ACC GTC ATT GTC AT
Fabp1	TCT CTT GCT GAC TCT CTT GTA GAC
	CTT TGA GCC ATT CAT GAA GGC AAT
Fabp2	ACA TCA GCT TAG CTC TTC AGC GTT
	ATG TGA TGA AGA GGA AGC TTG GAG
FHL1	TCG TGC CAG GAT TGT CCT TCA TAG
	CTG GCA TAA AGA CTG CTT CAC CTG
Fzd2	AGC AGG CGG CCG GGC AGC TA
	GTG GAA CTG GGC CGG TCC G
GAPDH	TaqMan mouse endogenous control (Applied Biosystems)
Hlx1	ACA GAC ATA CAA GCG GAA GCG CT
	TTA GAG TGC CGC CAC TTC ATC CT
Hmgcs2	CTT CCT GCG ATG CAT CTC ATC C
	CCA CTC TGC CCA AGA ATT GGC T
Lama5	GGCCAGGAAGAACCAGCTA
	GCAATCTTCTCACTGGTCTCG
NPY	ACCCTCGCTCTATCTCTGCTC
	CGTTTTCTGTGCTTTCCTTCA
PBGD	AAC CCT TGT GAT GCT GTT GTC
	GAC GAT GGC ACT GAA TTC CT
Pitx2	CCA GCA AGG AAA GAA TGA GGA TGT
	ACT CG GGC TTC CGT AAG GTT GG
Sfrp2	CCA AGG TGT GTG AAG CCT GCA AA
	GGA GAT GCG CTT GAA CTC TCT CT
Wnt10b	Quiagen Gene ID 22410
