# Supplementary material for: Abnormal Wnt and PI3Kinase Signaling in the Malformed Intestine of lama5 Deficient Mice
Source: PLoS One. 2012 May 30;7(5):e37710. doi: 10.1371/journal.pone.0037710 (PMC3364287; doi:10.1371/journal.pone.0037710)
Supplement: Methods S1 — Supplementary materials and methods. (DOC) [file pone.0037710.s007.doc]

**Methods S1**

**RNA extraction**

Samples were immediately stored in RNA Later (Ambion, Courtaboeuf, France). RNA was extracted using Tri Reagent (Molecular Research Center Inc., Euromedex, France) and further purified by an additional phenol/chloroform 5/1 (V/V) extraction. The integrity and purity of each RNA sample was checked by microcapillary electrophoresis using the Agilent 2100 Bioanalyser (Agilent Technologies, Massy, France).

**cDNA microarray preparation and data analysis**

The microarrays used for the transcriptome analysis contained 10,752 murine cDNA clones obtained from five different cDNA bank sources, corresponding to 2150 genes. Information on the different banks can be found in Benhamouche et al. (2006) [1]. Plasmid templates for the 10,752 clones were isolated from bacterial clones and inserts were amplified by PCR. The PCR products were checked on agarose gels and were purified by filtration on 96-well MAFB NOB50 multiscreen-FB filter plates (Millipore, Bedford, MA, USA), dried by evaporation at room temperature in a speedvac® (SC210A, Savant Instruments, Holbrook, NY, USA), resuspended in 75 % formamide/25% milliQ water at a final concentration of 0.5 µg/µl and were incubated for 48 h at 4° C. The products were transferred to a 1536 well plate (#3950, Corning, NY, USA). A Microdgrid II robot (Genomics Solutions, Cambridge, UK) was used to print each product on ultraGAPS Coated Slides (# 40016, Corning Ltd) at 20-22° C, in 45% relative humidity. Arrays were dried 48 h in a dessicator; then DNA was cross-linked to the slide by UV irradiation (600 mJoules) in a UV2500 Stratalinker™ (Stratagene, La Jolla, CA, USA). For quality control, replicates of cDNA probes were spotted in non adjacent areas. The microarray experiments were performed in quadruple with independently prepared RNA from mutant and wild-type embryonic intestines at embryonic day 15.5 derived from 4 different litters. For each RNA sample, a dye-swap labeling and hybridization was performed using a common reference RNA sample corresponding to 15.5-day embryonic intestine of C57B1/6J mice. Cy3- and Cy5- labeled cDNA probes were prepared by reverse transcription, using SuperScriptII from Gibco-BRL (Invitrogen Cell Culture, France). They were then mixed together, purified and concentrated using the Microcon YM-30 filter (Millipore; Molsheim, France), and finally resuspended in hybridization solution: 50 % formamide, 4X SSC, 0.6 % SDS, 5X Denhardt solution, 0.25 mg/ml of mouse Cot-1 DNA, 1mg/ml salmon sperm DNA and 1mg/ml poly(dA). The slides were immersed in a prehybridization solution (50 % formamide, 4X SSC, 0.1 % SDS, 0.1 % BSA fraction V) for 30 min at 46° C before probe hybridization in a humid chamber overnight at 46° C. Prior scanning, three posthybridization washes were performed. Array scanning and data analysis was done as described in Ghate *et al* [2].

The Gene Cluster 3.0 and Java Treeview 0.9.6 programs were used for analyzing microarray data. The normalized ratio of RNA sample (control or mutant) to reference RNA (log2) was calculated, and this ratio was directly used for the comparison of gene expression in the different samples.

**Semi-quantitative and quantitative RT-PCR**

At least 3 paired intestinal control and knockout samples taken from distinct litters were used. Primer sequences are described in **Table S2**. Total RNA was treated with DNase I (Roche Diagnostics, Meylan, France). cDNA synthesis was done according to Turck *et al* [3], or by using the High Capacity cDNA RT kit (Applied Biosystems Inc, France).

Semi-quantitative PCR were performed with the Red’y Star Mix 2X (Eurogentec, Seraing, Belgium) using an iCycler apparatus (Bio-Rad, Marnes-la-Coquette, France). Control PCR was performed directly on RNA without reverse transcription. PCR fragments were analyzed by 3% (w/v) agarose gel electrophoresis using the gel Doc 1000 apparatus (Bio-Rad). The 36B4 ribosomal gene served as a standard for sample normalization.

For quantitative real-time PCR analysis, we used the LightCycler™ system (Roche Diagnostics, Meylan, France) or the 7500 real-time PCR system (Applied Biosystems Inc, France). PCR amplification was performed using the FastStart DNA Master Mix SYBR Green I (Roche Diagnostics, Meylan, France) or using the TaqMan® Gene Expression Master mix for GAPDH or the *Power* SYBR® Green PCR Master Mix (Applied Biosystems Inc, France). PCR efficiency (E) determined by serial dilution of a pool of cDNA was calculated by the slope of the regression line (*E* = 10^−1/slope^ -1) and was higher than 80%. The specificity of the PCR products was assessed by generating a melting curve. The comparative CT method (ΔΔCT method) was used to quantify the cDNA of interest relative to the PBGD (porphobilinogen desaminase) or the GAPDH references. Data are represented as ratios of mean values (+/- SEM).

**RNA *in situ* hybridization**

*In situ* hybridization of Msx1-labeled probe on frozen sections of E14.5 mouse embryos was performed as described in Gradwohl *et al* [4]. The Msx-1 antisense RNA probe (probe provided by Dr R. Hill, MRC Human Genetics Unit, Edinburgh, UK) was synthesized from a 880pb cDNA inserted in a pTZ19 vector.

**Cell culture**

Immortalized mouse intestinal m-IC_Cl2_ cells [5] were maintained in culture at 37° C in DMEM/Ham F12 medium (Invitrogen Cell Culture, France) supplemented with 2.4 g/l D-glucose (Sigma), 5 µg/ml transferrin (Sigma), 50nM dexamethasone (Sigma), 5 µg/ml insulin (Sigma), 30 nM sodium selenite (Sigma), 1 nM triiodothyronine (Sigma), 10 ng/ml epidermal growth factor, 2% fetal calf serum and 1 % peni-streptomycin. Populations of lentiviral shRNA m-IC_Cl2_ infected cells were maintained in medium containing 0.2µg/ml puromycin (Invitrogen, Cell culture, France). Mesenchymal primary cultures used until the third passage were established from embryonic intestinal tissue derived from wild-type or laminin α5 deficient mice after collagenase treatment as described previously [6]. Muscle-derived primary cell cultures were obtained from adult small intestine as follows: the outer muscle layer was removed, minced with scissors and then incubated in a collagenase/trypsin solution (collagenase XI, Sigma, 300 U/ml; trypsin, Calbiochem, 0.1 mg/ml in RPMI medium) for 1 hour at 37° C under shaking. After centrifugation the cells were transferred to collagen-I coated dishes in RPMI medium (Invitrogen Cell Culture, France) supplemented with 10% fetal calf serum and gentamycin. These primary muscle cells were used until the 5^th^ passage. The human embryonic cell line HEK293 was maintained in DMEM medium (Invitrogen Cell Culture, France) supplemented with 10% fetal calf serum and gentamycin.

**Western blot analysis**

The western blot procedure is performed using standard protocol and is described in Turck *et al* [7]. The following antibodies were used: rabbit polyclonal antibodies against Akt (9272, Cell Signaling; 1:1000), Akt2 (1:500; kindly provided by Dr B.A Hemmings, Friedrich Miescher Institute, Basel; Yang *et al.* 2003), rabbit monoclonal antibodies to PTEN (138G6, Cell Signaling; 1:1000), mouse monoclonal antibodies against Phospho-Akt (4051, Ser473, Cell Signaling; 1:1000), actin (Mab 1501R, Chemicon; 1:15,000). After incubation with goat anti-rabbit or anti-mouse alkaline phosphatase-linked secondary antibodies, the blots were developed using ECL (enhanced chemilumiscence substrate, Amersham Life Science Ltd, Buckinghamshire, England). Signals were quantified by using the Quantity One software (BioRad, CA, USA). The MagicMark Western Standard (Invitrogen, Cergy Pointoise, France) was used as molecular weight marker.

**Detection of apoptosis and migration assays**

For survival assays, mouse m-IC_Cl2_ intestinal cells were cultured on uncoated dishes overnight in serum-free medium. After trypsinization, cells were allowed to attach on uncoated or laminin-511 coated dishes (15x10^4^ cells/ 0.7 cm^2^) for 6 h prior to 1 hour-treatment with 200 µM H_2_O_2_ in DMEM/HamF12 serum-deprived medium with or without 1.5µM wortmannin. Surviving cells were detected after 24 h with MTS (CellTiter 96^R^ AQueous One solution Cell Proliferation Assay; Promega, Madison, WI) as previously described [8]. Data are presented as percentage of recovered cells in absence of H_2_O_2_. Alternatively, cells undergoing apoptosis were detected by immunostaining of cleaved caspase-3 using rabbit polyclonal antibodies (RD Systems; diluted at 1:500). Four independent experiments were performed in duplicate.

Two types of cell migration assays were used. In order to follow the random (chemokinetic) migration of the cells, time-lapse sequence videomicroscopy was used. 2.5x10^5^ cells/cm^2^ were grown on plastic, laminin-111, laminin-511 enriched matrix or laminin-511 plus wortmaninn (final concentration of 1.5µM) for up to 48h. Cultures were maintained at 37°C, 5% CO_2_ in the humidified chamber of the inverted microscope (Axiovert 200, Zeiss) equipped with a digital camera (Coolsnap fx, Roper Scientific). Images were taken every 10 minutes and movies were reconstructed using the Metaview software (Universal Imaging). Migration velocity was determined for at least 20 cells on all different substrata. Results are presented as the averaged velocity expressed in µm/h. Cumulative migrated distance was determined for individual cells and compared for the different substrata. At the end point of the experiment, cells were fixed (in 4% paraformaldehyde for 30 mn), and stained with Phalloidin-FITC (Sigma P5282; 1/100) and DAPI. To assess the directed migration 2.5x10^5^ cells/9.6 cm^2^ were plated on uncoated, on laminin-111, and on laminin-511 coated dishes (with or without wortmaninn), immediately fixed in a 20 degree upright angle for 3 h before the dishes were horizontally placed to start the migration assay. The cell front was marked before and after the experiment at 24h. In both assays, mitomycin C (Sigma, 5µg/ml) was added to block cell proliferation.

**Plasmids, transfection experiments, and TCF/β-catenin reporter assays**

Cells were transfected with the TOPflash vector, a β-catenin/TCF/LEF-responsive Firefly luciferase reporter plasmid along with the Renilla luciferase reporter plasmid (pRL-null vector, Promega, France) used as an internal transfection control. Positive controls consisted of cells co-transfected with the TOPflash plasmid along with expression plasmids encoding TCF4 and mutated active β-catenin. The FOPflash vector in which the TCF-responsive elements are mutated was used as a negative control. Transfections were performed 24h after plating using JetPEI^TM^ reagent (Polyplus-Transfection, Illkirch, France) for the HEK293 cells or with the X-tremeGENE HP DNA Transfection Reagent (Roche, Penzberg, Germany) for the m-ICc_l2_ cells according to the manufacturer’s instructions. Luciferase firefly activity was normalized to luciferase Renilla activity.

**Immunostaining**

For indirect immunofluorescence, human or dissected mouse intestines were embedded in Tissue-Tek (Sakura, Labonord) and frozen on dry ice. Immunodetection of laminin α5 chain was performed on cryosections of human samples with the mouse monoclonal 4C7 antibodies (hybridoma obtained from Dr E. Engvall, The Burham Institute, La Jolla, CA). Immunodection of β1 and β4 integrin and Lutheran was performed on cryosections of unfixed whole E14-E15 embryos or mouse intestines using rat monoclonal antibodies CD29 (clone 9EG7; BD Pharmingen), 346-11A (provided by Dr Kennel, Oak Ridge, TN) and rabbit polyclonal anti-Lutheran antibody (454, provided by Dr Le Van Kim, Inserm U665, Paris; [9]). Detection of MyoD1, α smooth muscle actin and laminin α5 chain was performed on cultured mouse intestinal mesenchymal or adult smooth muscle cells. Cells were prefixed 10 min in 1% paraformaldehyde and permeabilized 10 min with 0.5 % Triton X-100 before incubation with mouse NCL-MyoD1 monoclonal (Novocastra; 1:20), α-smooth muscle actin (Sigma; 1:400) or polyclonal laminin α5 (kindly provided by Dr L. Sorokin, Münster, Germany) antibody. Bound antibodies were visualized with anti-mouse (Biorad), anti-rabbit (Nordic Immunological laboratories) or anti-rat (Jackson Laboratory) secondary antibody conjugated with fluorescein isothiocyanate. For actin detection in m-ICc_l2_ cells, glass coverslips were first coated with laminin-111 or recombinant laminin-511 (BioLamina AB, Sweden). After adhesion, cells were fixed 2% paraformaldehyde (10 mn) and directly incubated with TRITC-phalloidinin (Sigma; 1:200) in PBS/0.1 % Triton X-100 for 20min. DAPI was used to visualize nuclei. After mounting in a glycerol/PBS/phenylenediamine solution, sections or cells were examined using an epifluorescence microscope (AX 60, Olympus Optical Co, Hamburg, Germany). Pictures were taken with an Olympus digital camera. Cells were observed by laser scanning confocal microscopy (Leica TCS SP2).

Detection of MyoD by immunhistochemistry was performed on prefixed (4% paraformaldehyde, 2h) and deparaffined sections. Antigen-antibody complexes were detected by using the Vectastain ABC Kit (Vector Laboratories).

**References for the Materials and Methods S1**

1. Benhamouche S, Decaens T, Godard C, Chambrey R, Rickman DS et al. (2006) Apc tumor suppressor gene is the "Zonation-Keeper" of mouse liver. Developmental Cell 10: 759-770.

2. Ghate A, Befort K, Becker JA, Filliol D, Bole-Feysot C et al. (2007) Identification of novel striatal genes by expression profiling in adult mouse brain. Neuroscience 146: 1182-1192.

3. Turck N, Lefebvre O, Gross I, Gendry P, Kedinger M et al. (2006) Effect of laminin-1 on intestinal cell differentiation involves inhibition of nuclear nucleolin. J Cell Physiol 206: 545-555.

4. Gradwohl G, Fode C, Guillemot F (1996) Restricted expression of a novel murineatonal-related bHLH protein in undifferentiated neural precursors. Dev Biol 180: 227-241.

5. Bens M, Bogdanova A, Cluzeaud F, Miquerol L, Kerneis S et al. (1996) Transimmortalized mouse intestinal cells (m-ICcl2) that maintain a crypt phenotype. Am J Physiol -Cell Physiol 39: C1666-C1674.

6. Olsen J, Lefebvre O, Fritsch C, Troelsen JT, Orian-Rousseau V et al. (2000) Involvement of activator protein 1 complexes in the epithelium-specific activation of the laminin g2-chain gene promoter by hepatocyte growth factor (scatter factor). Biochem J 347: 407-417.

7. Turck N, Richert S, Gendry P, Stutzmann J, Kedinger M et al. (2004) Proteomic analysis of nuclear proteins from proliferative and differentiated human colonic intestinal epithelial cells. Proteomics 4: 93-105.

8. Turck N, Gross I, Gendry P, Stutzmann J, Freund JN et al. (2005) Laminin isoforms: biological roles and effects on the intracellular distribution of nuclear proteins in intestinal epithelial cells. Exp Cell Res 303: 494-503.

9. Rahuel C, Filipe A, Ritie L, El Nemer W, Patey-Mariaud N et al. (2008) Genetic inactivation of the laminin {alpha}5 chain receptor Lu/BCAM leads to kidney and intestinal abnormalities in the mouse. Am J Physiol Renal Physiol 294: F393-F406.
